# Discovery of a Novel Multitarget Analgesic Through an In Vivo-Guided Approach

**DOI:** 10.3390/ph18020205

**Published:** 2025-02-03

**Authors:** Guo Zhen, Nayeon Do, Nguyen Van Manh, Hee-Jin Ha, Hee Kim, Hyunsoo Kim, Kwanghyun Choi, Jihyae Ann, Jeewoo Lee

**Affiliations:** 1 College of Pharmacy, Seoul National University, Seoul 08826, Republic of Korea; gpiaoliang@163.com (G.Z.); nydoh@snu.ac.kr (N.D.); manhvan238@gmail.com (N.V.M.); 2 Medifron DBT, Seoul 08502, Republic of Korea; hjha@n-dic.com (H.-J.H.); neuro100@naver.com (H.K.); temps99@naver.com (H.K.); 93photon@naver.com (K.C.)

**Keywords:** novel analgesic, multitarget analgesic, in vivo-guided approach

## Abstract

**Background:** Pain is a complex condition influenced by peripheral, central, immune, and psychological factors. Multitarget approaches offer a more effective and safer alternative to single-target analgesics by enhancing efficacy, reducing side effects, and minimizing tolerance. This study aimed to identify a novel multitarget analgesic with improved pharmacological properties. **Methods:** An in vivo-guided screening approach was used to discover a new analgesic compound. Compound 29, derived from a novel scaffold inspired by opiranserin and vilazodone pharmacophores, was identified through analog screening in the formalin test. Its efficacy was further evaluated in the spinal nerve ligation (SNL) model of neuropathic pain. Mechanistic studies explored its interaction with neurotransmitter transporters and receptors, while pharmacokinetic and safety assessments were conducted to determine its stability, brain penetration, and potential toxicity. **Results:** Compound 29 demonstrated high potency in the formalin test, with an ED50 of 0.78 mg/kg in the second phase and a concentration-dependent effect in the first phase. In the SNL model, it produced dose-dependent analgesic effects, increasing withdrawal thresholds by 24% and 45% maximum possible effect (MPE) at 50 and 100 mg/kg, respectively. Mechanistic studies revealed strong triple uptake inhibition, particularly at dopamine (DAT) and serotonin (SERT) transporters, alongside high-affinity 5-HT2A receptor antagonism. Pharmacokinetic analysis indicated enhanced stability and blood–brain barrier permeability. In vitro studies confirmed its nontoxicity to HT-22 cells but revealed potential hERG inhibition and strong CYP3A4 inhibition. **Conclusions:** Compound 29 is a promising multitarget analgesic with potent efficacy and favorable pharmacokinetics. Ongoing optimization efforts aim to mitigate side effects and enhance its therapeutic profile for clinical application.

## 1. Introduction

Pain is a complex and multifactorial experience influenced by a combination of peripheral and central mechanisms, immune responses, and psychological factors [1]. This complexity presents a major challenge for the development of effective analgesic therapies, particularly for chronic and neuropathic pain conditions, which are often refractory to conventional treatments. Traditional single-target drugs, such as nonsteroidal anti-inflammatory drugs (NSAIDs) and opioids, have provided relief for some patients but are frequently associated with significant side effects, including tolerance, dependence, and limited efficacy in addressing the multiple dimensions of pain signaling.

In recent years, a shift toward multitarget approaches has gained momentum in drug discovery. By simultaneously modulating multiple pathways involved in pain transmission and modulation [2,3,4], multitarget analgesics offer a more comprehensive strategy that holds promise for better therapeutic outcomes [5,6,7]. While this approach has the potential to enhance efficacy and mitigate the risk of tolerance commonly associated with single-target drugs, it is important to consider that targeting multiple pathways could also increase the likelihood of adverse effects due to interactions with additional biological systems. A balanced evaluation of these potential benefits and risks is essential for the development of multitarget analgesics.

One promising approach to discovering novel multitarget analgesics is the use of in vivo-guided models, which allow for the real-time evaluation of drug candidates in biological systems that closely mimic human pain pathways [8,9,10]. In vivo models provide invaluable insights into the pharmacodynamics and pharmacokinetics of compounds, enabling the identification of promising drug candidates that engage multiple targets relevant to pain.In this study, we utilized an in vivo-guided approach to identify a novel multitarget analgesic that modulates key mechanisms involved in pain perception, transmission, and modulation. Through the integration of in vivo pain models and biochemical assays, we aimed to discover and characterize a compound that addresses the limitations of current analgesics and offers potential as a more effective treatment for chronic and neuropathic pain.

**Figure 1:**
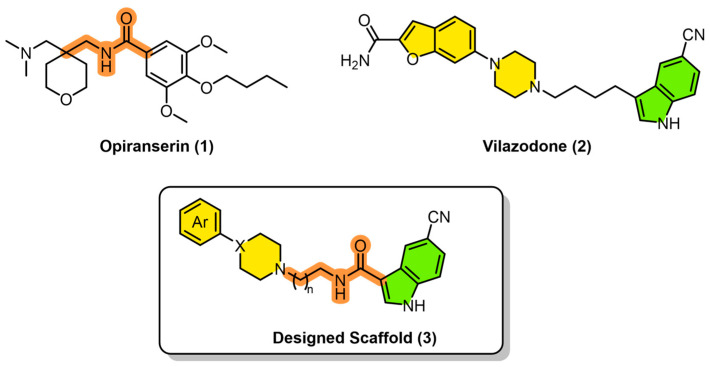
Newly designed scaffold based on the lead compounds.

Opiranserin (**1**) (Figure 1), also known as VVZ-149, is a multitarget analgesic identified through ex vivo screening [5]. This compound exhibits a unique pharmacological profile by selectively targeting several key pathways implicated in pain modulation. It functions as a glycine transporter 2 (GlyT2) blocker with an IC_50_ = 0.86 µM, effectively inhibiting the reuptake of glycine, a critical inhibitory neurotransmitter involved in spinal pain processing. Additionally, it acts as a purine receptor P2X_3_ antagonist with an IC_50_ = 0.87 µM, targeting receptors associated with nociceptive signaling in peripheral sensory neurons. Furthermore, it demonstrates antagonistic activity at the serotonin receptor 5-HT_2A_ with an IC_50_ = 1.3 µM, modulating central serotonin pathways that contribute to pain perception. This multitarget profile makes opiranserin a promising candidate for managing diverse pain types, offering an alternative to traditional analgesics. Opiranserin has been studied clinically for intravenous use in the management of postoperative pain, aiming to reduce the need for opioid [11]. The drug was approved on December 2024 by the Ministry of Food and Drug Safety in Korea for the short-term treatment of moderate and severe acute postoperative pain in adults.

Vilazodone (**2**) (Figure 1), primarily known as an antidepressant due to its dual action as a selective serotonin reuptake inhibitor (SSRI) and a partial agonist of the serotonin 5-HT_1A_ receptor, has shown potential as our lead compound in analgesic development [12,13]. Its mechanism of action, which modulates serotonin pathways, is relevant in pain perception and modulation. Serotonin plays a key role in descending pain pathways, which are involved in pain inhibition. By enhancing serotonin level and activating 5-HT_1A_ receptors, vilazodone may help alleviate pain, particularly in conditions where central sensitization and neuropathic pain are involved. Additionally, its dual action reduces the likelihood of developing side effects commonly associated with traditional analgesics. Therefore, vilazodone represents a promising candidate for the development of novel analgesics targeting pain-related pathways.

As a primary in vivo screening method, we employed the formalin test, a widely used experimental model in pain research, particularly for assessing the efficacy of potential analgesic candidates [14,15,16,17,18]. It involves injecting formalin into the paw of an animal, which induces a biphasic pain response—initial acute pain (first phase) followed by a prolonged inflammatory phase (second phase). This biphasic response allows researchers to evaluate analgesics that target both nociceptive and persistent inflammatory pain mechanisms, offering insights into the multitarget potential of a compound. Since the test engages both peripheral nociceptor activation and central sensitization mechanisms, it enables the screening of compounds that may work at multiple levels of the pain pathway. Analgesics that reduce both the first and second phases of the formalin test could be considered as targeting both peripheral and central pain mechanisms**.** Given the complexity of the pain response in the formalin test, it is an ideal model for assessing multitarget analgesics. A novel analgesic may show efficacy in modulating both inflammatory and neuropathic components of pain, thus having broader therapeutic potential.

In our efforts to discover a novel multitarget analgesic through in vivo-guided screening, we designed a new scaffold (**3**) (Figure 1) inspired by the pharmacophores of opiranserin and vilazodone. This design integrates a 5-cyanoindole core and either arylpiperazine or arylpiperidine, both of which are well-known 5-HT receptor modulators. These components are connected via an alkylamido linker, enabling dual functionality [19].

In this study, a series of scaffold **3** analogs was synthesized and subsequently screened for their ability to inhibit the second phase of the formalin test, a key model for assessing analgesic potential. Among the synthesized compounds, compound **29** emerged as the lead candidate due to its significant inhibitory activity. This compound was selected for further in-depth investigation, including studies in a neuropathic pain model, detailed mechanism of action analyses, and comprehensive pharmacokinetic and toxicological evaluations.

## 2. Results and Discussion

### 2.1. Synthesis

**Scheme 1:**
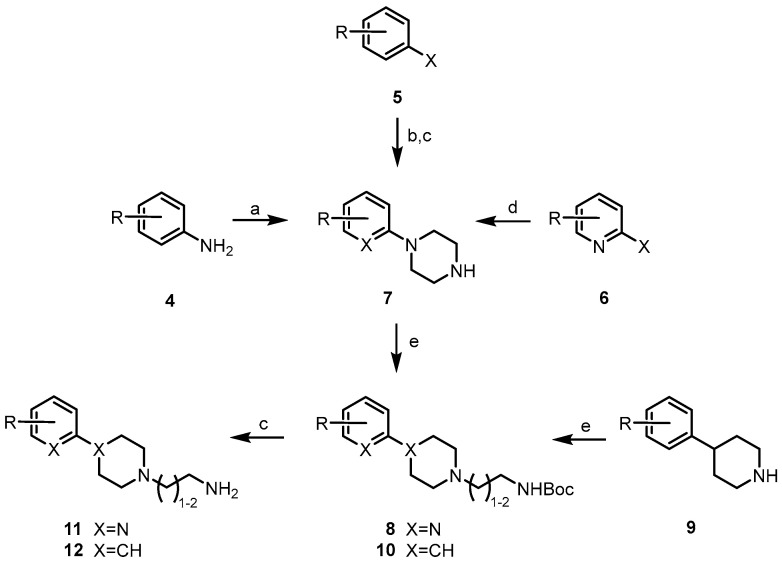
Synthesis of 4-aryl-1-aminoalkylpiperazine/piperidine analogs. Reagents and conditions: (a) [Method A] HCl∙NH(CH_2_CH_2_Cl)_2_, 1,2-dichlorobenzene, 180 °C, 2 h: [Method B] HCl∙NH(CH_2_CH_2_Cl)_2_, ethylene glycol monomethyl ether, 150 °C, overnight; (b) *N*-Boc-piperazine, Pd_2_(dba)_3_, NaOtBu, BINAP, toluene, reflux, overnight; (c) CF_3_CO_2_H, CH_2_Cl_2_, r.t., 1 h; (d) piperazine, ethylene glycol, 140 °C, 2 h; (e) I(CH_2_)_2_NHBoc or Br(CH_2_)_3_NHBoc, CH_3_CN, 60 °C, overnight.

For the synthesis of 4-aryl-1-aminoalkylpiperazine (Scheme 1), 1-arylpiperazine **7**, a key intermediate was prepared using three distinct methods. First, an aniline-type aryl **4** was reacted directly with bis(2-chloroethyl)amine hydrochloride to produce piperazine **7**. Second, aromatic halides **5** underwent palladium-catalyzed coupling with *N*-Boc-piperazine, followed by Boc group deprotection, yielding **7**. Third, a 2-chloropyridine-type aryl **6** was directly condensed with piperazine to obtain **7**. Subsequent alkylation of **7** with (*N*-Boc)-aminoethyl iodide or (*N*-Boc)-aminopropyl bromide produced intermediate **8**, which, upon Boc group deprotection, yielded 1-aryl-4-aminoalkylpiperidine **11**.

Conversely, 4-aryl-1-aminoalkylpiperidine **12** was synthesized starting from commercially available 4-arylpiperidine **9**, following the same synthetic pathway employed for the corresponding piperazine analogs.

**Scheme 2:**
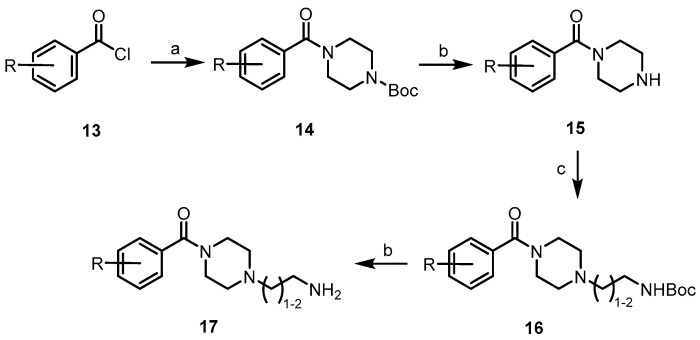
Synthesis of 4-arylcarbonyl-1-aminoalkylpiperazine. Reagents and conditions: (a) *N*-Boc-piperazine, NEt_3_, CH_2_Cl_2_, r.t., 3 h; (b) CF_3_CO_2_H, CH_2_Cl_2_, r.t., 1 h; (c) I(CH_2_)_2_NHBoc or Br(CH_2_)_3_NHBoc, CH_3_CN, 60 °C, overnight.

For the synthesis of 4-arylcarbonyl-1-aminoalkylpiperazine (Scheme 2), aromatic acyl halide **13** was condensed with *N*-Boc-piperazine to form an intermediate **14**, which, after Boc deprotection, yielded **15**. Alkylation of **15** with (*N*-Boc)-aminoalkyl halide, followed by subsequent deprotection provided the primary amines **17**.

**Scheme 3:**
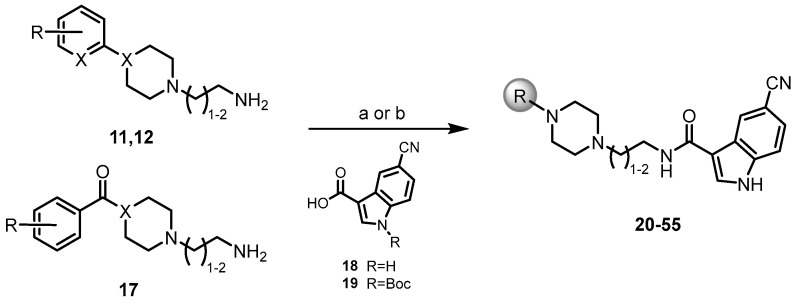
Synthesis of the final compounds. Reagents and conditions: (a) **18**, EDC, HOBt, DIPEA, CH_2_C1_2_, r.t., overnight; (b) (i) **19**, EDC, HOBt, DIPEA, CH_2_C1_2_, r.t., overnight, (ii) CF_3_CO_2_H, CH_2_Cl_2_, r.t., 6 h.

The final compounds (**20**–**55**) were synthesized by coupling the amines, **11**, **12**, and **17**, with either 5-cyano-1*H*-indole-3-carboxylic acid **18** or its *N*-Boc protected analog **19**, using 1-ethyl-3-(3-dimethylaminopropyl)carbodiimide (EDC) as the coupling reagent (Scheme 3). This synthetic strategy enabled the generation of a diverse library of compounds for pharmacological evaluation.

### 2.2. Animal Studies

#### 2.2.1. Primary In Vivo Screening

For primary screening, the synthesized compounds were administered to mice via intraperitoneal injection at a dose of 5 mg/kg using the formalin model. The percentage inhibition in the second phase, relative to the vehicle control (DMSO/cremophor EL/distilled water = 10:10:80), was calculated. The results are presented in Table 1 and Table 2, and are compared to gabapentin, which was used as a positive control and demonstrated 70.7% inhibition at 5 mg/kg.

The structure–activity relationship (SAR) analysis provided valuable insights. In the series of piperazine analogs, the ethyl linker derivatives generally demonstrated greater inhibition compared to the corresponding propyl linker derivatives (Table 1). Within the aryl ring series, the 3-substituted phenyl analogs (**20**–**22**, **36**–**38**) exhibited superior inhibitory activity compared to the corresponding 4-substituted analogs (**23**–**25**, **39**–**41**). Notably, 3-chlorophenyl analogs, **21** and **37**, as well as the 3-trifluoromethyl analog **22**, showed exceptional inhibition in this test. In contrast, the pyridine analogs (**26**–**28**, **42**–**44**) displayed moderate inhibitory activity.

Further investigation into various bicyclic aryl groups commonly utilized in 5-HT modulating CNS drugs revealed additional trends. Examples include quinoline (**29**, **45**) as seen in quipazine; 2-carbamoylbenzofuran (**30**, **46**) as in vilazodone; (2,4-dimethylphenyl)thiophenyl (**31**, **47**) as in vortioxetine; benzisothiazole (**32**, **48**) as in ziprasidone. Among these, only quinoline analogs, **29** and **45**, demonstrated strong inhibition in both ethyl and propyl linkers. Additionally, arylcarbonyl analogs (**33**–**35**, **49**–**51**), including 2,3-dihydrobenzo [1,4]dioxine-2-carbonyl group as in doxazosin, exhibited a weak to moderate inhibitory activity.

In the series of piperidine analogs, derivatives such as 6-fluorobenzoisoxazole (**52**, **54**) as in risperidone, and 6-chlorobenzimidazol-2-one (**53**, **55**) as in clopimozide, were explored at the 4-position of piperidine. These compounds also displayed weak to moderate inhibitory activity (Table 2).

Overall, six compounds, 3-chlorophenyl (**21** and **37**), 3-trifluorophenyl (**22**), quinoline (**29** and **45**), (2,4-dimethylphenyl)thiophenyl (**31**) derivatives, exhibited inhibition percentages exceeding 70% among the 36 compounds tested. These compounds were selected for further evaluation to determine their ED_50_ values in the formalin model.

#### 2.2.2. Formalin Model Study

The six selected compounds, which demonstrated strong inhibitory effects exceeding 70% during initial screenings, were further evaluated to determine their ED_50_ values in the second phase of the formalin model. Each compound was assessed using four doses (0.1, 1, 5, and 10 mg/kg) by intraperitoneal injection.

**Figure 2:**
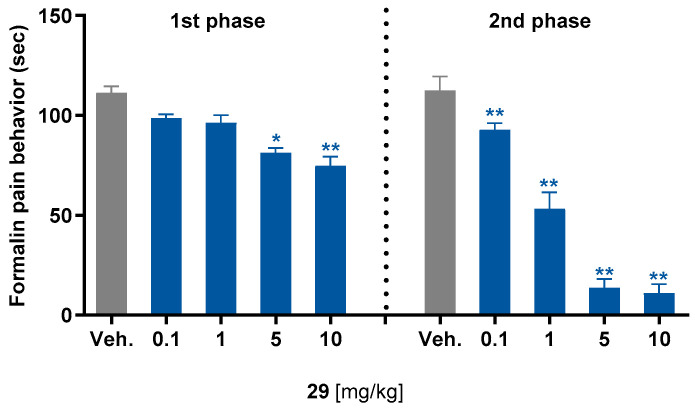
Analgesic activity of compound **29** in the formalin model. Results are expressed as mean ± SEM (*n* = 8). * *p* < 0.05, ** *p* < 0.01 compared to the vehicle, based on one-way analysis of variance (ANOVA), followed by Bonferroni’s post hoc test.

All tested compounds exhibited potent analgesic effects, with ED_50_ values ranging from 0.79 to 3.2 mg/kg in the second phase of the model. Among them, compound **29** demonstrated the highest potency, with an ED_50_ value of 0.78 mg/kg (Table 3). While the activity of the compound in the first phase was relatively weak, significant alterations indicative of concentration-dependent inhibition was observed during this phase (Figure 2). This finding suggests that compound **29** may exert its effects through mechanisms that are more prominent in the later, inflammatory phase of the formalin model. Such results underscore the compound’s potential as a highly effective analgesic agent with both dose-dependent and phase-specific activity.

#### 2.2.3. Neuropathic Pain Model Study

To evaluate its potency in neuropathic pain, compound **29** was tested in the spinal nerve ligation (SNL) model, a representative model for neuropathic pain [20,21].

The SNL model, also known as the Chung model, is a widely used animal model for studying neuropathic pain, a chronic pain condition resulting from nervous system damage. This model replicates human neuropathic pain, which can stem from nerve injuries due to conditions like diabetes, shingles, or spinal cord injury. In the SNL model, the L5 or L6 spinal nerve is surgically ligated to induce nerve injury, leading to the development of long-lasting neuropathic pain symptoms in the animal. Following the nerve injury, animals display pain-related behaviors that mirror those seen in human neuropathic pain conditions, such as allodynia and hyperalgesia**.** Behavioral assessments conducted 14 days after surgery evaluate the animal’s sensitivity to mechanical stimuli using Von Frey filaments to quantify pain responses and measure mechanical allodynia.

**Figure 3:**
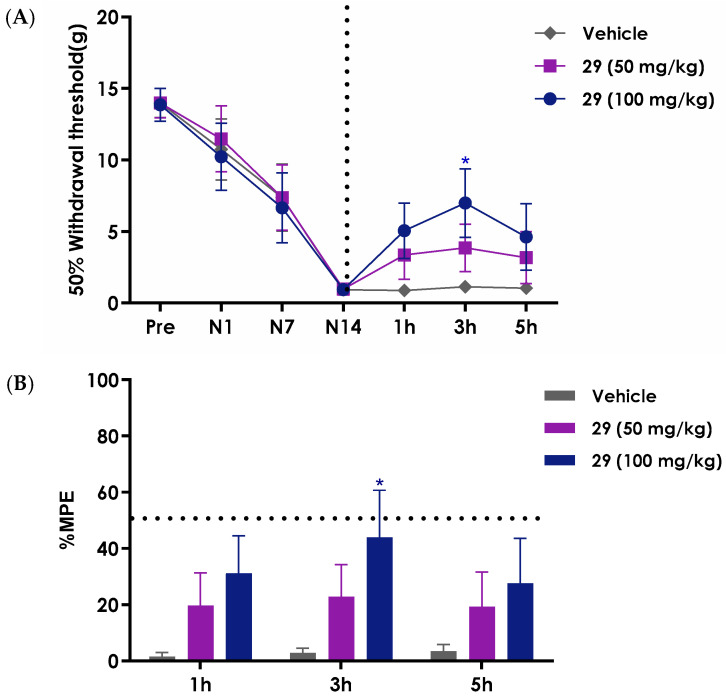
Antinociceptive effect of compound 29 on mechanical allodynia in SNL model rats. (**A**) The mechanical allodynia was induced by spinal nerve ligation and evaluated by measuring paw withdrawal thresholds in response to von Frey filament stimuli. Results are expressed as 50% of paw withdrawal thresholds measured before surgery and at 1, 7, and 14 days post-ligation (N1, N7, and N14), as well as at 1, 3, and 5 h following administration of either the vehicle or compound 29 (50 and 100 mg/kg). The dotted line indicates the initiation of treatment with compound 29. (**B**) The antinociceptive effects of compound 29 (50 and 100 mg/kg) at 1, 3, and 5 h post-administration are shown as the percentage of the maximum possible effect (MPE). The MPE was calculated using the following formula: MPE (%) = [(post-treated value − pre-treatment threshold) × 100]/(maximum possible threshold − pre-treatment threshold). Data are expressed as mean ± SEM. Statistical significance was determined using Student’s *t*-test: * *p* < 0.05 compared to the vehicle-treated group.

In this experiment, compound **29** was administered intraperitoneally at two different doses, 50 and 100 mg/kg, and its analgesic effects were assessed by measuring the 50% withdrawal threshold at multiple time points (1, 3, and 5 h post-administration) (Figure 3A). Compound **29** demonstrated dose-dependent analgesic activity, as evidenced by an increase in the withdrawal threshold. The analgesic effect was most pronounced at 3 h after administration, indicating a T_max_ of 3 h, as confirmed by an in vivo pharmacokinetic study. At this peak, the compound achieved a maximum possible effect (MPE) of 24% at the 50 mg/kg dose and 45% at the 100 mg/kg dose (Figure 3B). These results clearly highlight the compound’s ability to produce significant analgesic effects in a dose-dependent manner in a neuropathic pain model, with a notable enhancement in efficacy observed at the higher dose.

### 2.3. Mechanism Study

To elucidate the mechanism of action of compound **29** in producing analgesic effects, its inhibitory effects were systematically evaluated through a screening process. The compound was tested at a concentration of 10 μM against a diverse panel of 47 drug target molecules that are closely associated with pain modulation pathways. The results indicated that, among them, 16 targets exhibited more than 50% inhibition in response to compound **29**. This suggests that the compound interacts significantly with a subset of targets involved in pain signaling. To further refine these findings and understand the potency of its activity, IC_50_ values were subsequently determined for those receptors, compared to their corresponding representative reference, demonstrating the highest levels of inhibition among the 16 identified targets (Table 4).

Compound **29** demonstrated strong inhibition of monoamine reuptake at dopamine (DAT), norepinephrine (NET), and serotonin (SERT) transporters, with K_i_ values of 0.046, 0.38, and 0.13 µM, respectively. These values indicate that it was 19 times more potent than at DAT compared to vanoxerine, 3 times more potent at NET compared to desipramine, and 13 times more potent at SERT compared to fluoxetine, highlighting compound **29**’s superior efficacy in modulating monoamine transporter activity compared to these widely known reference compounds.

Additionally, compound **29** exhibited notable binding affinity for several key receptors associated with pain modulation and neurotransmitter systems. Specifically, it showed binding affinities for the adrenergic a_2A_ receptor, dopamine D_2L_ (long isoform of D_2_ receptor), and serotonin 5-HT_2A_ receptor, with K_i_ values of 4.11, 1.85, and 0.15 µM, respectively. These values correspond to 0.25, 0.02, and 1.53 times the activity of reference compounds, yohimbine, spiperone, and ketanserin, respectively, highlighting the compound’s broad interaction with the neurotransmitter system.

Overall, the analgesic mechanism of compound **29** is primarily attributed to its strong triple monoamine uptake inhibition, with a particularly potent effect observed at DAT and SERT. Additionally, its activity at the 5-HT_2A_ receptor further supports its potential as a multifaceted agent in pain management. In fact, several multitarget analgesics currently used in clinical practice, including tramadol, duloxetine, venlafaxine, amitriptyline, and tapentadol, share a similar mechanism of action to compound **29**, involving SERT and NET inhibition. However, the direct involvement of these mechanisms in the observed antinociceptive effects has not been conclusively demonstrated and requires further investigation. The combined activity across these targets underscores the compound’s promise as a therapeutic candidate with a diverse and complementary mechanism of action.

### 2.4. Pharmacokinetic Study

In the in vitro metabolic stability study, the stability of compound **29** was evaluated using liver microsomes derived from both mouse and human sources. The primary objectives of this study were to determine the metabolic clearance rate of compound **29** over a defined time period, enabling an assessment of its intrinsic clearance. Microsomal incubations were performed, and the percentage of intact compound remaining was quantified at specific time points.

After 30 min of incubation, it was observed that 51.2% of the parent compound remained intact in mouse liver microsomes, while 55.6% remained intact in human liver microsomes (Table 5). These results suggest that compound **29** demonstrates moderate metabolic stability across both species, with slightly higher stability in human microsomes compared to mouse microsomes. Such findings are indicative of the compound’s potential for further investigation in drug development.

To assess the ability of compound **29** to permeate the blood–brain barrier (BBB), a BBB PAMPA (Parallel Artificial Membrane Permeability Assay) experiment was conducted. This assay is a widely used in vitro model to predict the potential of compounds to cross the BBB. During the experiment, the permeability of compound **29** was quantified by measuring its LogPe value, which was determined to be 4.5 (Table 5). This high value strongly indicates that it has a high potential to permeate the BBB efficiently. With its high BBB permeability, compound **29** is expected to exert pharmacological effects in the brain, making it a promising candidate for central nervous system (CNS) drugs.

**Figure 4:**
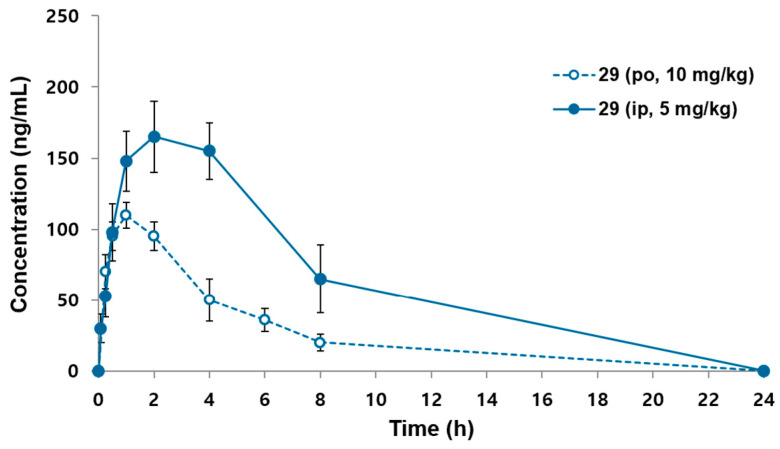
Mean plasma concentration–time profile of compound **29** following intraperitoneal and oral administration in Sprague–Dawley rats.

The in vivo pharmacokinetics parameters of compound **29** were measured in rats over 24 h following intraperitoneal (IP) and oral (PO) administration (Figure 4). These experiments were conducted to evaluate the compound’s absorption, distribution, metabolism, and excretion profiles under different routes of administration.

Following IP administration at a dose of 5 mg/kg, the mean area under the curve from the time of dosing to the last measurable concentration (AUC_last_) was 1030 ng·h/mL, with a maximum plasma concentration (C_max_) of 208 ng/mL. The half-life (t_1/2_) of the compound under this route of administration was calculated to be 3.49 h, with a time to reach maximum concentration (T_max_) of 2.83 h. Apparent clearance (Cl/F) and volume of distribution (V_d_) were determined to be 3.88 L/h/kg and 17.9 L/kg, respectively. These results suggest a relatively high systemic exposure and sustained plasma concentration following IP administration.

In contrast, oral administration at a dose of 10 mg/kg resulted in an AUC_last_ of 407 ng·h/mL and a C_max_ of 113 ng/mL. The half-life was significantly shorter at 1.67 h, and the T_max_ was observed to be 1.50 h. Cl/F and V_d_ were determined to be 24.04 L/h/kg and 57.4 L/kg, respectively, indicating high clearance and extensive distribution into the compartment beyond the plasma compared to IP treatment. Compared to IP administration, the oral route showed approximately a five-fold lower exposure and two-fold lower in vivo stability (Table 6).

These findings highlight the pharmacokinetic advantages of IP administration for animal studies of compound **29**, demonstrating greater systemic exposure and prolonged stability compared to oral administration.

### 2.5. Toxicity Study

In the in vitro toxicity study, we initially evaluated the cytotoxic potential of compound **29** using a hippocampal neuronal cell line (HT-22), a commonly used model for assessing neurotoxicity. Cell viability was determined after exposure to compound **29** at a concentration of 10 μM. The result indicated that it did not exhibit any toxic effects on HT-22 cells, as cell viability was maintained at levels exceeding 100% relative to the untreated control group. These findings suggest that compound **29** is nontoxic at the tested concentration, supporting its potential for further development and use in biological systems without significant cytotoxicity concerns.

The hERG gene encodes a cardiac potassium ion channel (Kv11.1) that is critical for the repolarization phase of the cardiac action potential. This channel plays a pivotal role in returning ventricular muscle cells to their resting state following depolarization. Inhibition or suppression of hERG channel activity by a drug can lead to a prolonged action potential duration, increasing the risk of cardiac arrhythmias such as torsades de pointes, which may progress to the more fatal condition of ventricular fibrillation.

To evaluate the cardiotoxicity of compound **29**, a hERG fluorescence polarization assay was conducted, with results compared to E-4031, a well-known and potent hERG channel inhibitor used as a positive reference. At a concentration of 10 μM, it exhibited 52.7% inhibition of hERG channel activity. Based on this degree of inhibition, its IC_50_ value was estimated to be approximately 10 μM (Table 7). These findings indicate that compound **29** has a moderate potential for hERG channel inhibition, which warrants further investigation to assess its cardiotoxic risk in clinical applications.

In the drug–drug interaction study, the potential inhibitory effects of compound **29** on cytochrome P450 (CYP) enzymes were assessed by examining five representative isozymes: CYP1A2, CYP3A4, CYP2C9, CYP2C19, and CYP2D6. Ketoconazole, a well-characterized strong inhibitor of CYP3A4, was used as the reference inhibitor to validate the assay conditions. At a tested concentration of 10 μM, compound **29** exhibited minimal inhibitory activity (less than 50% inhibition) against CYP1A2, CYP2C9, CYP2C19, and CYP2D6, indicating a low likelihood of significant interactions with these enzymes. However, it demonstrated substantial inhibition of CYP3A4, the most abundant isozyme, with 78% activity suppression under the same conditions. This level of inhibition suggests that compound **29** is a strong CYP3A4 inhibitor, which may have implications for its potential drug–drug interaction profile (Table 8).

## 3. Materials and Methods

### 3.1. Chemistry

All chemical reagents and solvents were commercially available. Silica gel column chromatography was performed using ZEOprep 60/40–63 μm silica gel (ZEOCHEM, Louisville, KY, USA). 1H and 13C NMR spectra were recorded on a JEOL JNM-ECZ400S spectrometer (400 MHz for 1H and 100 MHz for 13C; JEOL Ltd., Akishima, Tokyo, Japan).

Chemical shifts are reported in parts per million (ppm) relative to tetramethylsilane (Me_4_Si) as the internal standard. High-resolution mass spectra (HRMS) were measured by fast atom bombardment (FAB) with a JEOL JMS-700 MStation instrument (JEOL Ltd., Akishima, Tokyo, Japan). All final compounds were purified to greater than 95% purity, as determined by high-performance liquid chromatography (HPLC). HPLC was performed on an Agilent 1120 Compact LC (G4288A) instrument (Agilent Technologies, Santa Clara, CA, USA) using an Agilent TC-C18 column (4.6 mm × 250 mm, 5 μm).

**General Procedure**

#### 3.1.1. Synthesis of 1-Arylpiperazine **7** (Procedure 1)

*Method A*: A mixture of aniline-type aryl **4** (1.0 g, 1.0 equiv) and bis(2-chloroethyl)amine hydrochloride (1.2 equiv) in 1,2-dichlorobenzene was stirred at 180 °C for 2 h, or alternatively, in ethylene glycol monomethyl ether at 150 °C overnight. Upon completion, the reaction mixture was cooled to room temperature, diluted with water, and extracted twice with ethyl acetate (EtOAc). The combined organic layers were washed three times with water, dried over MgSO_4_, and concentrated under reduced pressure. The resulting residue was purified via silica gel column chromatography using methanol (MeOH)/methylene chloride (MC) mixtures (1:20 and 1:10) as eluents to obtain the desired product **7**.

*Method B*, *C*: A solution of aromatic halides **5** (500 mg, 1.0 equiv) in toluene was treated with NaOtBu (2.0 equiv), Pd_2_(dba)3 (0.025 equiv), BINAP (0.05 equiv), and N-Boc-piperazine (1.0 equiv) and refluxed overnight. Upon completion, the reaction mixture was cooled to room temperature, diluted with water, and extracted twice with EtOAc. The combined organic layers were washed three times with water, dried over MgSO_4_, and concentrated under reduced pressure. The resulting residue was purified via silica gel column chromatography using MeOH/MC (1:20 and 1:10) as eluents to afford the Boc-piperazine intermediate. The intermediate (200 mg, 1.0 equiv) was dissolved in MC and treated with trifluoroacetic acid (TFA) (10 equiv) at 0 °C. The reaction was stirred at room temperature for 1 h. Upon completion, the reaction mixture was neutralized to pH > 7 with 1N NaOH at 0 °C, diluted with water, and extracted twice with MC and EtOAc. The combined organic layers were dried over MgSO_4_ and concentrated under reduced pressure to afford the desired product **7**.

*Method D*: A mixture of 2-chloropyridine-type aryl **6** (500 mg, 1.0 equiv) and piperazine (10.0 equiv) in ethylene glycol was stirred at 140 °C for 2 h. Upon completion, the reaction mixture was cooled to room temperature, diluted with water, and extracted twice with EtOAc. The combined organic layers were washed three times with water, dried over MgSO_4_, and concentrated under reduced pressure. The resulting residue was purified by silica gel column chromatography using MeOH/MC (1:20 and 1:10) as eluents to obtain the desired product **7**.

#### 3.1.2. Alkylation of **7** or **9** or **15** (Procedure 2)

A solution of 1-arylpiperazine **7**, commercially available 4-arylpiperidine **9**, or intermediate **15** (200 mg, 1.0 equiv) in acetonitrile (ACN) was treated with (*N*-Boc)-aminoethyl iodide or (*N*-Boc)-aminopropyl bromide (1.5 equiv) and K_2_CO_3_ (2.0 equiv) and stirred at 60 °C overnight. Upon completion, the reaction mixture was cooled to room temperature, filtered to remove K_2_CO_3_, washed with MC, and concentrated under reduced pressure. The residue was purified by silica gel column chromatography using MeOH/MC (1:40) as the eluent to afford the desired product **8**, **10**, or **16**.

#### 3.1.3. Deprotection of **8** or **10** or **14** or **16** (Procedure 3)

A solution of intermediate **8**, **10**, **14**, or **16** (200 mg, 1.0 equiv) in MC was treated with TFA (10.0 equiv) at 0 °C and stirred at room temperature for 1 h. Upon completion, the reaction mixture was neutralized to pH > 7 with 1N NaOH at 0 °C, diluted with water, and extracted twice with MC and EtOAc. The combined organic layers were dried over MgSO_4_ and concentrated under reduced pressure to afford the desired product **11**, **12**, **15**, or **17**.

#### 3.1.4. Synthesis of **14** (Procedure 4)

A mixture of aromatic acyl halide **13** (500 mg, 1.0 equiv) in MC was combined with *N*-Boc-piperazine (1.0 equiv) and triethylamine (TEA) (2.0 equiv) at 0 °C and stirred at room temperature for 3 h. Upon completion, the reaction mixture was diluted with water and extracted twice with MC. The combined organic layers were dried over MgSO_4_ and concentrated under reduced pressure. The resulting residue was purified by silica gel column chromatography using EtOAc/hexane (1:7 and 1:4) as eluents to obtain the desired product **14**.

#### 3.1.5. EDC Coupling (Procedure 5)

A mixture of 5-cyano-1*H*-indole-3-carboxylic acid **18** or its N-Boc protected analog **19** (0.9 equiv) in MC was treated with HOBt (1.5 equiv), EDC (1.5 equiv), and DIPEA (3.0 equiv) at 0 °C and stirred for 30 min. Subsequently, amine **11**, **12**, or **17** (100 mg, 1.0 equiv) was added, and the reaction mixture was stirred at room temperature overnight. Upon completion, the reaction mixture was diluted with water and extracted twice with MC and EtOAc. The combined organic layers were dried over MgSO_4_ and concentrated under reduced pressure. The resulting residue was purified by silica gel column chromatography using MeOH/MC (1:20) as the eluent to obtain the desired products **20**–**55**.

**5-Cyano-*N*-(2-(4-(3-fluorophenyl)piperazin-1-yl)ethyl)-1*H*-indole-3-carboxamide (20)**

Yield 55%, white solid, mp: 225.8 °C; ^1^H NMR (500 MHz, DMSO-*d*_6_) δ 12.01 (s, 1H), 8.46–8.54 (m, 1H), 8.15–8.22 (m, 1H), 7.96–8.02 (m, 1H), 7.57–7.63 (m, 1H), 7.42–7.51 (m, 1H), 7.06–7.18 (m, 1H), 6.66–6.74 (m, 2H), 6.47–6.54 (m, 1H), 3.40 (q, *J* = 6.6 Hz, 2H), 3.13–3.15 (m, 4H), 2.53–2.59 (m, 4H), 2.50 (t, *J* = 6.9 Hz, 2H); ^13^C NMR (500 MHz, DMSO-*d*_6_) δ 164.93, 164.19, 162.86, 153.38, 153.31, 138.40, 130.83, 130.75, 130.62, 126.83, 126.35, 125.13, 120.96, 113.91, 111.96, 111.31, 111.29, 105.15, 104.99, 103.13, 102.28, 102.08, 57.77, 53.13, 48.20, 36.67; HRMS (FAB) calc. for C_22_H_22_FN_5_O, [M+H]^+^: 392.1887, found: 392.1898.

***N*-(2-(4-(3-Chlorophenyl)piperazin-1-yl)ethyl)-5-cyano-1*H*-indole-3-carboxamide (21)**

Yield 50%, white solid, mp: 218.2 °C; ^1^H NMR (500 MHz, DMSO-*d*_6_) δ 12.01 (s, 1H), 8.51–8.63 (m, 1H), 8.14–8.17 (m, 1H), 7.96–8.02 (m, 1H), 7.57–7.71 (m, 1H), 7.45–7.51 (m, 1H), 7.13–7.20 (m, 1H), 6.87–6.98 (m, 1H), 6.84–6.86 (m, 1H), 6.72–6.76 (m, 1H), 3.37–3.47 (m, 2H), 3.10–3.20 (m, 4H), 2.53–2.60 (m, 4H), 2.50 (t, *J* = 7.1 Hz, 2H); ^13^C NMR (500 MHz, DMSO-*d*_6_) δ 164.19, 152.80, 138.40, 134.32, 130.91, 130.63, 126.83, 126.36, 125.13, 120.97, 118.47, 114.99, 114.12, 113.91, 111.96, 103.13, 57.77, 53.14, 48.19, 36.67; HRMS (FAB) calc. for C_22_H_22_ClN_5_O, [M+H]^+^: 408.1591, found: 408.1596.

**5-Cyano-*N*-(2-(4-(3-(trifluoromethyl)phenyl)piperazin-1-yl)ethyl)-1*H*-indole-3-carboxamide (22)**

Yield 17%, white solid, mp: 214.7 °C; ^1^H NMR (500 MHz, DMSO-*d*_6_) δ 12.03 (s, 1H), 8.51 (t, *J* = 0.8 Hz, 1H), 8.14 (s, 1H), 8.01 (t, *J* = 5.7 Hz, 1H), 7.58 (dd, *J* = 8.5, 0.6 Hz, 1H), 7.47 (dd, *J* = 8.5, 1.6 Hz, 1H), 7.37 (t, *J* = 8.0 Hz, 1H), 7.18 (d, *J* = 8.5 Hz, 1H), 7.12 (s, 1H), 7.02 (d, *J* = 7.5 Hz, 1H), 3.40 (q, *J* = 6.5 Hz, 2H), 3.20 (t, *J* = 5.0 Hz, 4H), 2.56–2.60 (m, 4H), 2.51 (t, *J* = 6.9 Hz, 2H); ^13^C NMR (500 MHz, DMSO-*d*_6_) δ 164.19, 151.77, 138.34, 130.63, 130.50, 130.47, 126.82, 126.35, 126.06, 125.13, 120.96, 119.09, 115.00, 113.97, 111.96, 111.16, 103.12, 57.75, 53.13, 48.14, 36.57; HRMS (FAB) calc. for C_23_H_22_F_3_N_5_O, [M+H]^+^: 442.1855, found: 442.1848.

**5-Cyano-*N*-(2-(4-(4-fluorophenyl)piperazin-1-yl)ethyl)-1*H*-indole-3-carboxamide (23)**

Yield 68%, white solid, mp: 222.6 °C; ^1^H NMR (500 MHz, DMSO-*d*_6_) δ 11.90–12.02 (m, 1H), 8.47–8.54 (m, 1H), 8.10–8.17 (m, 1H), 7.96–8.02 (m, 1H), 7.55–7.60 (m, 1H), 7.44–7.50 (m, 1H), 6.96–7.04 (m, 2H), 6.83–6.93 (m, 2H), 3.39 (q, *J* = 6.6 Hz, 2H), 3.00–3.05 (m, 4H), 2.55–2.60 (m, 4H), 2.50 (t, *J* = 6.9 Hz, 2H); ^13^C NMR (500 MHz, DMSO-*d*_6_) δ 164.22, 157.41, 155.54, 148.48, 138.40, 130.62, 126.82, 126.34, 125.14, 120.97, 117.59, 117.53, 115.83, 115.65, 113.91, 111.96, 103.13, 57.77, 53.31, 49.51, 36.67; HRMS (FAB) calc. for C_22_H_22_FN_5_O, [M+H]^+^: 392.1887, found: 392.1883.

***N*-(2-(4-(4-Chlorophenyl)piperazin-1-yl)ethyl)-5-cyano-1*H*-indole-3-carboxamide (24)**

Yield 71%, white solid, mp: 252.1 °C; ^1^H NMR (500 MHz, DMSO-*d*_6_) δ 12.02 (s, 1H), 8.47–8.55 (m, 1H), 8.14 (s, 1H), 8.01 (t, *J* = 5.8 Hz, 1H), 7.54–7.60 (m, 1H), 7.41–7.49 (m, 1H), 7.13–7.21 (m, 2H), 6.85–6.96 (m, 2H), 3.39 (q, *J* = 6.5 Hz, 2H), 3.05–3.10 (m, 4H), 2.55 (t, *J* = 5.0 Hz, 4H), 2.50 (t, *J* = 7.1 Hz, 2H); ^13^C NMR (500 MHz, DMSO-*d*_6_) δ 164.22, 150.37, 138.40, 130.62, 129.10, 126.82, 126.34, 125.14, 122.76, 120.97, 117.29, 113.91, 111.95, 103.13, 57.76, 53.15, 48.54, 36.66; HRMS (FAB) calc. for C_22_H_22_ClN_5_O, [M+H]^+^: 408.1591, found: 408.1596.

**5-Cyano-*N*-(2-(4-(4-(trifluoromethyl)phenyl)piperazin-1-yl)ethyl)-1*H*-indole-3-carboxamide (25)**

Yield 62%, white solid, mp: 250.9 °C; ^1^H NMR (500 MHz, DMSO-*d*_6_) δ 12.02 (s, 1H), 8.51 (q, *J* = 0.7 Hz, 1H), 8.16 (d, *J* = 15.1 Hz, 1H), 7.96–8.02 (m, 1H), 7.53–7.59 (m, 1H), 7.44–7.48 (m, 3H), 7.01 (d, *J* = 8.8 Hz, 2H), 3.40 (q, *J* = 6.5 Hz, 2H), 3.21–3.26 (m, 4H), 2.54–2.60 (m, 4H), 2.51 (t, *J* = 6.9 Hz, 2H); ^13^C NMR (500 MHz, DMSO-*d*_6_) δ 164.20, 153.81, 138.40, 130.63, 126.83, 126.68, 126.64, 126.61, 126.36, 125.13, 120.96, 118.15, 114.63, 113.91, 111.96, 103.13, 57.76, 53.02, 47.53, 36.66; HRMS (FAB) calc. for C_23_H_22_F_3_N_5_O, [M+H]^+^: 442.1855, found: 442.1848.

**5-Cyano-*N*-(2-(4-(6-(trifluoromethyl)pyridin-2-yl)piperazin-1-yl)ethyl)-1*H*-indole-3-carboxamide (26)**

Yield 67%, white solid, mp: 231.3 °C; ^1^H NMR (500 MHz, DMSO-*d*_6_) δ 12.02 (s, 1H), 8.51–8.54 (m, 1H), 8.17 (d, *J* = 14.4 Hz, 1H), 7.96–8.03 (m, 1H), 7.63–7.71 (m, 1H), 7.55–7.60 (m, 1H), 7.42–7.51 (m, 1H), 7.07–7.11 (m, 1H), 7.00 (q, *J* = 7.2 Hz, 1H), 3.52 (t, *J* = 4.9 Hz, 4H), 3.40 (q, *J* = 6.5 Hz, 2H), 2.48–2.58 (m, 6H); ^13^C NMR (500 MHz, DMSO-*d*_6_) δ 164.22, 159.07, 145.25, 144.98, 139.51, 138.40, 130.63, 126.84, 126.36, 125.13, 123.28, 120.96, 113.89, 111.96, 111.22, 109.10, 109.07, 103.13, 57.79, 52.95, 44.84, 36.66; HRMS (FAB) calc. for C_22_H_21_F_3_N_6_O, [M+H]^+^: 443.1807, found: 443.1804.

***N*-(2-(4-(3-Chloropyridin-2-yl)piperazin-1-yl)ethyl)-5-cyano-1*H*-indole-3-carboxamide (27)**

Yield 90%, white solid, mp: 243.3 °C; ^1^H NMR (500 MHz, DMSO-*d*_6_) δ 12.02 (s, 1H), 8.51 (d, *J* = 1.6 Hz, 1H), 8.15–8.20 (m, 2H), 8.01 (s, 1H), 7.75 (dd, *J* = 7.5, 1.6 Hz, 1H), 7.58 (dd, *J* = 8.5, 0.6 Hz, 1H), 7.47 (dd, *J* = 8.5, 1.9 Hz, 1H), 6.95 (dd, *J* = 7.8, 4.7 Hz, 1H), 3.39–3.45 (m, 2H), 3.24 (s, 4H), 2.62 (d, *J* = 36.4 Hz, 4H), 2.48–2.52 (m, 2H); ^13^C NMR (500 MHz, DMSO-*d*_6_) δ 164.23, 158.27, 146.55, 139.59, 138.40, 130.63, 126.84, 126.37, 125.13, 122.05, 120.96, 118.88, 113.91, 111.88, 103.13, 57.87, 53.22, 49.27, 36.65; HRMS (FAB) calc. for C_21_H_21_ClN_6_O, [M+H]^+^: 409.1544, found: 409.1548.

**5-Cyano-*N*-(2-(4-(3-(trifluoromethyl)pyridin-2-yl)piperazin-1-yl)ethyl)-1*H*-indole-3-carboxamide (28)**

Yield 49%, white solid, mp: 222.9 °C; ^1^H NMR (500 MHz, DMSO-*d*_6_) δ 12.02 (s, 1H), 8.50 (d, *J* = 0.9 Hz, 1H), 8.46 (dd, *J* = 4.7, 1.3 Hz, 1H), 8.15–8.17 (m, 1H), 7.99–8.02 (m, 2H), 7.57–7.60 (m, 1H), 7.47 (dd, *J* = 8.5, 1.6 Hz, 1H), 7.12 (ddd, *J* = 7.7, 4.9, 0.8 Hz, 1H), 3.39 (q, *J* = 6.6 Hz, 2H), 3.18 (t, *J* = 4.7 Hz, 4H), 2.55–2.65 (m, 4H), 2.50 (q, *J* = 7.1 Hz, 2H); ^13^C NMR (500 MHz, DMSO-*d*_6_) δ 164.24, 159.57, 152.09, 138.40, 138.23, 138.20, 130.61, 126.83, 126.36, 125.14, 120.96, 117.96, 115.79, 115.54, 113.91, 111.93, 103.13, 57.81, 53.32, 50.95, 36.66; HRMS (FAB) calc. for C_22_H_21_F_3_N_6_O, [M+H]^+^: 443.1807, found: 443.1814.

**5-Cyano-*N*-(2-(4-(quinolin-2-yl)piperazin-1-yl)ethyl)-1*H*-indole-3-carboxamide (29)**

Yield 72%, white solid, mp: 228.2 °C; ^1^H NMR (500 MHz, DMSO-*d*_6_) δ 12.02 (s, 1H), 8.52–8.55 (m, 1H), 8.17 (d, *J* = 14.4 Hz, 1H), 8.03 (t, *J* = 5.7 Hz, 1H), 7.99 (d, *J* = 9.1 Hz, 1H), 7.64–7.69 (m, 1H), 7.57–7.62 (m, 1H), 7.52 (d, *J* = 7.8 Hz, 1H), 7.37–7.49 (m, 2H), 7.16–7.24 (m, 2H), 3.66 (t, *J* = 4.9 Hz, 4H), 3.38–3.47 (m, 2H), 2.48–2.60 (m, 6H); ^13^C NMR (500 MHz, DMSO-*d*_6_) δ 164.22, 157.61, 147.78, 138.41, 137.84, 130.64, 129.88, 127.89, 126.85, 126.55, 126.37, 125.13, 123.26, 122.53, 120.97, 113.91, 111.97, 110.63, 103.13, 57.88, 53.31, 45.15, 36.70; HRMS (FAB) calc. for C_25_H_24_N_6_O, [M+H]^+^: 425.2090, found: 425.2102.

***N*-(2-(4-(2-Carbamoylbenzofuran-5-yl)piperazin-1-yl)ethyl)-5-cyano-1*H*-indole-3-carboxamide (30)**

Yield 60%, white solid, mp: 258.4 °C; ^1^H NMR (500 MHz, DMSO-*d*_6_) δ 12.01 (s, 1H), 8.51 (t, *J* = 0.8 Hz, 1H), 8.15 (s, 1H), 7.97–8.02 (m, 2H), 7.58 (td, *J* = 4.2, 0.7 Hz, 1H), 7.53 (t, *J* = 9.1 Hz, 1H), 7.47 (dd, *J* = 8.5, 1.6 Hz, 1H), 7.43 (dd, *J* = 9.7, 0.9 Hz, 1H), 7.35–7.36 (m, 1H), 7.12–7.15 (m, 2H), 3.37–3.47 (m, 2H), 3.07–3.16 (m, 4H), 2.59–2.64 (m, 4H), 2.52 (t, *J* = 6.9 Hz, 2H); ^13^C NMR (500 MHz, DMSO-*d*_6_) δ 164.19, 160.42, 150.20, 149.62, 148.86, 138.40, 130.63, 128.26, 126.83, 126.35, 125.14, 120.97, 118.60, 113.92, 112.32, 111.97, 110.19, 108.07, 103.13, 57.82, 53.47, 50.34, 36.71; HRMS (FAB) calc. for C_25_H_24_N_6_O_3_, [M+H]^+^: 457.1988, found: 457.1974.

**5-Cyano-*N*-(2-(4-(2-((2,4-dimethylphenyl)thio)phenyl)piperazin-1-yl)ethyl)-1*H*-indole-3-carboxamide (31)**

Yield 65%, white solid, mp: 247.2 °C; ^1^H NMR (500 MHz, DMSO-*d*_6_) δ 12.02 (d, *J* = 1.9 Hz, 1H), 8.52 (d, *J* = 1.6 Hz, 1H), 8.16 (d, *J* = 2.8 Hz, 1H), 8.01 (t, *J* = 5.5 Hz, 1H), 7.57–7.61 (m, 1H), 7.48 (dd, *J* = 8.5, 1.6 Hz, 1H), 7.29 (d, *J* = 7.8 Hz, 1H), 7.20 (d, *J* = 12.2 Hz, 1H), 7.10 (dd, *J* = 8.0, 1.4 Hz, 1H), 7.04–7.07 (m, 2H), 6.84–6.87 (m, 1H), 6.34 (dd, *J* = 7.8, 1.3 Hz, 1H), 3.39–3.47 (m, 2H), 2.98 (s, 4H), 2.60 (s, 4H), 2.54 (t, *J* = 6.6 Hz, 2H), 2.28–2.32 (m, 3H), 2.16–2.23 (m, 3H); ^13^C NMR (500 MHz, DMSO-*d*_6_) δ 164.20, 149.64, 142.19, 139.60, 138.41, 136.28, 133.87, 132.20, 130.65, 128.52, 127.93, 126.83, 126.34, 126.14, 125.14, 124.75, 120.99, 120.59, 113.92, 112.00, 103.13, 57.85, 53.72, 51.78, 36.72, 21.24, 20.63; HRMS (FAB) calc. for C_30_H_31_N_5_OS, [M+H]^+^: 510.2328, found: 510.2317.

***N*-(2-(4-(Benzo[d]isothiazol-3-yl)piperazin-1-yl)ethyl)-5-cyano-1*H*-indole-3-carboxamide (32)**

Yield 52%, white solid, mp: 256.7 °C; ^1^H NMR (500 MHz, DMSO-*d*_6_) δ 12.03 (d, *J* = 1.9 Hz, 1H), 8.52–8.55 (m, 1H), 8.18 (dd, *J* = 14.8, 2.8 Hz, 1H), 8.04 (t, *J* = 5.5 Hz, 1H), 8.00–8.02 (m, 2H), 7.56–7.62 (m, 1H), 7.50–7.54 (m, 1H), 7.44–7.48 (m, 1H), 7.38–7.42 (m, 1H), 3.42 (t, *J* = 6.4 Hz, 6H), 2.74 (d, *J* = 63.4 Hz, 4H), 2.48–2.58 (m, 2H); ^13^C NMR (500 MHz, DMSO-*d*_6_) δ 164.27, 164.04, 152.52, 138.41, 130.65, 128.39, 127.88, 126.84, 126.37, 125.15, 124.93, 124.69, 121.58, 120.99, 113.92, 111.92, 103.14, 57.84, 53.11, 50.15, 36.64; HRMS (FAB) calc. for C_23_H_22_N_6_OS, [M+H]^+^: 431.1654, found: 431.1664.

**5-Cyano-*N*-(2-(4-(4-fluorobenzoyl)piperazin-1-yl)ethyl)-1*H*-indole-3-carboxamide (33)**

Yield 31%, white solid, mp: 148.1 °C; ^1^H NMR (500 MHz, DMSO-*d*_6_) δ 12.03 (s, 1H), 8.50 (d, *J* = 0.9 Hz, 1H), 8.14 (d, *J* = 2.3 Hz, 1H), 8.01 (t, *J* = 5.5 Hz, 1H), 7.57–7.66 (m, 1H), 7.47 (dd, *J* = 8.3, 1.4 Hz, 1H), 7.38–7.44 (m, 2H), 7.21–7.25 (m, 2H), 3.49–3.76 (m, 2H), 3.34–3.46 (m, 4H), 2.48–2.63 (m, 4H), 2.44–2.32 (2H); ^13^C NMR (500 MHz, DMSO-*d*_6_) δ 168.54, 164.19, 164.00, 162.05, 138.40, 132.88, 132.85, 130.62, 130.07, 129.99, 126.83, 126.34, 125.13, 120.97, 115.99, 115.81, 113.91, 111.95, 103.12, 57.58, 53.15, 42.29, 36.59; HRMS (FAB) calc. for C_23_H_22_FN_5_O_2_, [M+H]^+^: 420.1836, found: 420.1844.

**5-Cyano-*N*-(2-(4-(3-(trifluoromethyl)benzoyl)piperazin-1-yl)ethyl)-1*H*-indole-3-carboxamide (34)**

Yield 71%, white solid, mp: 143.5 °C; ^1^H NMR (500 MHz, DMSO-*d*_6_) δ 12.12 (d, *J* = 68.4 Hz, 1H), 8.51–8.68 (m, 1H), 8.15–8.19 (m, 1H), 7.97–8.09 (m, 1H), 7.54–7.69 (m, 1H), 7.46–7.53 (m, 1H), 7.24–7.42 (m, 1H), 7.18 (d, *J* = 8.5 Hz, 1H), 7.13 (d, *J* = 12.9 Hz, 1H), 7.01–7.05 (m, 1H), 3.38–3.47 (m, 2H), 3.20 (t, *J* = 4.9 Hz, 4H), 2.56–2.63 (m, 4H), 2.51 (t, *J* = 7.1 Hz, 2H); ^13^C NMR (500 MHz, DMSO-*d*_6_) δ 164.20, 151.77, 138.41, 130.64, 130.50, 130.46, 130.26, 126.83, 126.36, 126.07, 125.12, 123.91, 120.97, 119.18, 115.00, 113.91, 111.95, 111.31, 103.12, 57.75, 53.13, 48.14, 36.67; HRMS (FAB) calc. for C_24_H_22_F_3_N_5_O_2_, [M+H]^+^: 470.1804, found: 470.1817.

**5-Cyano-*N*-(2-(4-(2,3-dihydrobenzo[*b*][1,4]dioxine-2-carbonyl)piperazin-1-yl)ethyl)-1*H*-indole-3-carboxamide (35)**

Yield 83%, white solid, mp: 140.2 °C; ^1^H NMR (500 MHz, DMSO-*d*_6_) δ 12.02 (s, 1H), 8.51 (s, 1H), 8.15 (s, 1H), 7.96–8.01 (m, 1H), 7.58 (d, *J* = 8.5 Hz, 1H), 7.48 (dd, *J* = 8.5, 1.3 Hz, 1H), 6.84–6.86 (m, 1H), 6.78–6.83 (m, 3H), 5.17 (dd, *J* = 6.4, 2.4 Hz, 1H), 4.33 (dd, *J* = 11.9, 2.5 Hz, 1H), 4.13 (dd, *J* = 11.8, 6.4 Hz, 1H), 3.36–3.59 (m, 6H), 2.48–2.60 (m, 4H), 2.35 (d, *J* = 27.6 Hz, 2H); ^13^C NMR (500 MHz, DMSO-*d*_6_) δ 165.17, 164.20, 143.58, 143.39, 138.40, 130.63, 126.84, 126.35, 125.14, 121.97, 121.85, 120.97, 117.52, 117.38, 113.91, 111.95, 103.13, 69.91, 65.25, 57.58, 53.50, 52.95, 45.60, 42.00, 36.57; HRMS (FAB) calc. for C_25_H_25_N_5_O_4_, [M+H]^+^: 460.1985, found: 460.1987.

**5-Cyano-*N*-(3-(4-(3-fluorophenyl)piperazin-1-yl)propyl)-1*H*-indole-3-carboxamide (36)**

Yield 33%, white solid, mp: 229.9 °C; ^1^H NMR (500 MHz, DMSO-*d*_6_) δ 12.02 (s, 1H), 8.50 (t, *J* = 0.8 Hz, 1H), 8.14–8.17 (m, 1H), 7.96–8.06 (m, 1H), 7.57 (dd, *J* = 8.5, 0.6 Hz, 1H), 7.46 (dd, *J* = 8.5, 1.9 Hz, 1H), 7.16 (q, *J* = 8.0 Hz, 1H), 6.66–6.71 (m, 2H), 6.48 (td, *J* = 8.2, 2.3 Hz, 1H), 3.28 (q, *J* = 6.5 Hz, 2H), 3.12 (t, *J* = 5.0 Hz, 4H), 2.46 (q, *J* = 1.9 Hz, 4H), 2.32–2.38 (m, 2H), 1.66–1.75 (m, 3H); ^13^C NMR (500 MHz, DMSO-*d*_6_) δ 164.84, 164.24, 162.86, 153.34, 138.40, 130.83, 130.75, 130.54, 126.88, 126.44, 125.07, 121.00, 113.89, 111.97, 111.30, 111.28, 105.14, 104.98, 103.04, 102.26, 102.06, 56.16, 53.13, 48.21, 37.58, 27.22; HRMS (FAB) calc. for C_23_H_24_FN_5_O, [M+H]^+^: 406.2043, found: 406.2031.

***N*-(3-(4-(3-Chlorophenyl)piperazin-1-yl)propyl)-5-cyano-1*H*-indole-3-carboxamide (37)**

Yield 61%, white solid, mp: 240.1 °C; ^1^H NMR (500 MHz, DMSO-*d*_6_) δ 12.00 (s, 1H), 8.51–8.54 (m, 1H), 8.15 (d, *J* = 2.5 Hz, 1H), 7.96–8.06 (m, 1H), 7.56–7.59 (m, 1H), 7.46–7.50 (m, 1H), 7.14–7.18 (m, 1H), 6.88 (t, *J* = 2.2 Hz, 1H), 6.83–6.85 (m, 1H), 6.72–6.74 (m, 1H), 3.27 (t, *J* = 6.7 Hz, 2H), 3.11–3.13 (m, 4H), 2.46 (q, *J* = 1.9 Hz, 4H), 2.27–2.38 (m, 2H), 1.66–1.75 (m, 2H); ^13^C NMR (500 MHz, DMSO-*d*_6_) δ 164.23, 152.79, 138.39, 134.32, 130.91, 130.47, 126.90, 126.43, 125.11, 120.99, 118.46, 114.96, 114.09, 113.86, 111.99, 103.08, 56.15, 53.12, 48.18, 37.57, 27.21; HRMS (FAB) calc. for C_23_H_24_ClN_5_O, [M+H]^+^: 422.1748, found: 422.1739.

**5-Cyano-*N*-(3-(4-(3-(trifluoromethyl)phenyl)piperazin-1-yl)propyl)-1*H*-indole-3-carboxamide (38)**

Yield 27%, white solid, mp: 208.1 °C; ^1^H NMR (500 MHz, DMSO-*d*_6_) δ 12.00 (s, 1H), 8.51 (q, *J* = 0.7 Hz, 1H), 8.14 (d, *J* = 3.5 Hz, 1H), 8.05 (t, *J* = 5.5 Hz, 1H), 7.57 (dd, *J* = 8.5, 0.6 Hz, 1H), 7.47 (dd, *J* = 8.5, 1.6 Hz, 1H), 7.37 (t, *J* = 8.0 Hz, 1H), 7.17 (dd, *J* = 8.5, 2.5 Hz, 1H), 7.11 (s, 1H), 7.01 (d, *J* = 7.5 Hz, 1H), 3.27 (d, *J* = 7.2 Hz, 2H), 3.13–3.21 (m, 4H), 2.49 (t, *J* = 5.0 Hz, 4H), 2.37 (t, *J* = 7.1 Hz, 2H), 1.67–1.75 (m, 2H); ^13^C NMR (500 MHz, DMSO-*d*_6_) δ 164.23, 151.77, 138.39, 130.47, 130.26, 126.89, 126.42, 126.05, 125.11, 123.88, 120.99, 119.16, 115.02, 113.87, 111.99, 111.22, 103.07, 56.16, 53.12, 48.14, 37.58, 27.22; HRMS (FAB) calc. for C_24_H_24_F_3_N_5_O, [M+H]^+^: 456.2011, found: 456.2025.

**5-Cyano-*N*-(3-(4-(4-fluorophenyl)piperazin-1-yl)propyl)-1*H*-indole-3-carboxamide (39)**

Yield 34%, white solid, mp: 240.2 °C; ^1^H NMR (500 MHz, DMSO-*d*_6_) δ 12.14 (s, 1H), 8.51 (d, *J* = 1.6 Hz, 1H), 8.34 (s, 1H), 8.24 (d, *J* = 2.5 Hz, 1H), 7.59 (d, *J* = 8.5 Hz, 1H), 7.48 (dd, *J* = 8.5, 1.6 Hz, 1H), 7.03–7.15 (m, 2H), 6.97 (q, *J* = 4.5 Hz, 2H), 3.41–3.67 (m, 4H), 3.33 (t, *J* = 6.3 Hz, 2H), 3.05–3.11 (m, 6H), 1.90–2.04 (m, 2H); ^13^C NMR (500 MHz, DMSO-*d*_6_) δ 164.73, 138.42, 130.87, 126.82, 126.36, 125.17, 120.99, 118.35, 118.26, 116.09, 115.91, 113.95, 111.66, 103.15, 53.91, 42.20, 18.54, 17.25, 12.72; HRMS (FAB) calc. for C_23_H_24_FN_5_O, [M+H]^+^: 406.2043, found: 406.2039.

***N*-(3-(4-(4-Chlorophenyl)piperazin-1-yl)propyl)-5-cyano-1*H*-indole-3-carboxamide (40)**

Yield 19%, white solid, mp: 241.8 °C; ^1^H NMR (500 MHz, DMSO-*d*_6_) δ 12.00 (s, 1H), 8.50 (q, *J* = 0.8 Hz, 1H), 8.14 (s, 1H), 8.05 (t, *J* = 5.7 Hz, 1H), 7.57 (dd, *J* = 8.5, 0.6 Hz, 1H), 7.47 (dd, *J* = 8.5, 1.6 Hz, 1H), 7.16–7.19 (m, 2H), 6.89 (td, *J* = 6.4, 3.8 Hz, 2H), 3.24–3.28 (m, 2H), 3.07–3.12 (m, 4H), 2.47–2.48 (m, 4H), 2.32–2.37 (m, 2H), 1.66–1.74 (m, 2H); ^13^C NMR (500 MHz, DMSO-*d*_6_) δ 164.23, 150.38, 138.39, 130.47, 129.10, 126.89, 126.42, 125.11, 122.73, 120.99, 117.27, 113.87, 111.99, 103.07, 56.16, 53.15, 48.56, 37.58, 27.21; HRMS (FAB) calc. for C_23_H_24_ClN_5_O, [M+H]^+^: 422.1748, found: 422.1745.

**5-Cyano-*N*-(3-(4-(4-(trifluoromethyl)phenyl)piperazin-1-yl)propyl)-1*H*-indole-3-carboxamide (41)**

Yield 38%, white solid, mp: 250.7 °C; ^1^H NMR (500 MHz, DMSO-*d*_6_) δ 12.00 (s, 1H), 8.51 (d, *J* = 0.9 Hz, 1H), 8.15 (d, *J* = 2.5 Hz, 1H), 8.05 (t, *J* = 5.7 Hz, 1H), 7.57 (dd, *J* = 8.5, 0.9 Hz, 1H), 7.44–7.48 (m, 3H), 7.01 (d, *J* = 8.8 Hz, 2H), 3.26 (s, 2H), 3.23 (t, *J* = 5.0 Hz, 4H), 2.48 (t, *J* = 4.7 Hz, 4H), 2.32–2.38 (m, 2H), 1.67–1.75 (m, 2H); ^13^C NMR (500 MHz, DMSO-*d*_6_) δ 164.23, 153.81, 138.39, 130.47, 126.89, 126.68, 126.64, 126.42, 125.11, 120.99, 118.43, 118.14, 114.62, 113.87, 111.99, 103.08, 56.14, 53.01, 47.53, 37.55, 27.21; HRMS (FAB) calc. for C_24_H_24_F_3_N_5_O, [M+H]^+^: 456.2011, found: 456.2025.

**5-Cyano-*N*-(3-(4-(6-(trifluoromethyl)pyridin-2-yl)piperazin-1-yl)propyl)-1*H*-indole-3-carboxamide (42)**

Yield 82%, white solid, mp: 230.7 °C; ^1^H NMR (500 MHz, DMSO-*d*_6_) δ 12.02 (s, 1H), 8.51–8.54 (m, 1H), 8.15–8.19 (m, 1H), 8.02–8.07 (m, 1H), 7.67–7.76 (m, 1H), 7.53–7.61 (m, 1H), 7.43–7.51 (m, 1H), 7.06–7.11 (m, 1H), 6.98 (d, *J* = 7.2 Hz, 1H), 3.58 (d, *J* = 69.1 Hz, 4H), 3.27 (d, *J* = 7.2 Hz, 2H), 2.44 (s, 4H), 2.32–2.38 (m, 2H), 1.62–1.73 (m, 2H); ^13^C NMR (500 MHz, DMSO-*d*_6_) δ 164.24, 159.07, 144.94, 139.52, 138.39, 130.48, 126.90, 126.44, 125.09, 123.28, 120.99, 113.86, 111.97, 111.22, 109.29, 103.07, 56.18, 52.95, 44.81, 37.54, 27.03; HRMS (FAB) calc. for C_23_H_23_F_3_N_6_O, [M+H]^+^: 457.1964, found: 457.1977.

***N*-(3-(4-(3-Chloropyridin-2-yl)piperazin-1-yl)propyl)-5-cyano-1*H*-indole-3-carboxamide (43)**

Yield 93%, white solid, mp: 201.8 °C; ^1^H NMR (500 MHz, DMSO-*d*_6_) δ 12.02 (s, 1H), 8.51–8.54 (m, 1H), 8.15–8.21 (m, 2H), 7.99–8.08 (m, 1H), 7.73–7.77 (m, 1H), 7.53–7.61 (m, 1H), 7.42–7.49 (m, 1H), 6.93–6.98 (m, 1H), 3.23–3.27 (m, 6H), 2.51–2.77 (m, 4H), 2.32–2.40 (m, 2H), 1.58–1.73 (m, 2H); ^13^C NMR (500 MHz, DMSO-*d*_6_) δ 164.25, 158.23, 146.55, 139.58, 138.39, 130.48, 126.90, 126.44, 125.09, 122.05, 120.99, 118.95, 113.86, 111.98, 103.07, 56.19, 53.15, 49.20, 37.53, 26.99; HRMS (FAB) calc. for C_22_H_23_ClN_6_O, [M+H]^+^: 423.1700, found: 423.1696.

**5-Cyano-*N*-(3-(4-(3-(trifluoromethyl)pyridin-2-yl)piperazin-1-yl)propyl)-1*H*-indole-3-carboxamide (44)**

Yield 63%, white solid, mp: 201.9 °C; ^1^H NMR (500 MHz, DMSO-*d*_6_) δ 12.01 (s, 1H), 8.50–8.53 (m, 1H), 8.47 (dd, *J* = 4.7, 1.6 Hz, 1H), 8.15–8.18 (m, 1H), 8.05–8.10 (m, 1H), 7.96–8.01 (m, 1H), 7.57–7.61 (m, 1H), 7.47 (dd, *J* = 8.5, 1.6 Hz, 1H), 7.13 (dd, *J* = 7.7, 4.9 Hz, 1H), 3.27 (t, *J* = 6.4 Hz, 2H), 3.17 (t, *J* = 4.6 Hz, 4H), 2.51–2.65 (m, 4H), 2.32–2.41 (m, 2H), 1.67–1.75 (m, 2H); ^13^C NMR (500 MHz, DMSO-*d*_6_) δ 164.27, 159.55, 152.10, 138.39, 138.23, 138.19, 130.48, 126.89, 126.42, 125.58, 125.11, 123.38, 120.99, 118.04, 115.72, 113.87, 111.96, 103.08, 56.09, 53.22, 50.86, 37.50, 27.07; HRMS (FAB) calc. for C_23_H_23_F_3_N_6_O, [M+H]^+^: 457.1964, found: 457.1977.

**5-Cyano-*N*-(3-(4-(quinolin-2-yl)piperazin-1-yl)propyl)-1*H*-indole-3-carboxamide (45)**

Yield 44%, white solid, mp: 215.2 °C; ^1^H NMR (500 MHz, DMSO-*d*_6_) δ 12.07 (s, 1H), 8.52 (s, 1H), 8.17 (s, 1H), 8.13 (s, 1H), 8.00 (d, *J* = 9.2 Hz, 1H), 7.66 (d, *J* = 7.8 Hz, 1H), 7.58 (d, *J* = 8.3 Hz, 1H), 7.38–7.54 (m, 3H), 7.16–7.28 (m, 2H), 3.80 (d, *J* = 114.0 Hz, 4H), 3.46–3.34 (2H), 2.71 (d, *J* = 133.7 Hz, 4H), 2.03–2.29 (m, 2H), 1.74 (s, 2H); ^13^C NMR (500 MHz, DMSO-*d*_6_) δ 172.54, 164.32, 147.72, 138.41, 137.92, 130.55, 129.93, 127.91, 126.89, 126.56, 126.43, 125.12, 123.32, 122.64, 121.00, 113.89, 111.92, 110.64, 103.10, 56.14, 53.27, 44.78, 37.31, 21.59; HRMS (FAB) calc. for C_26_H_26_N_6_O, [M+H]^+^: 439.2246, found: 439.2248.

***N*-(3-(4-(2-Carbamoylbenzofuran-5-yl)piperazin-1-yl)propyl)-5-cyano-1*H*-indole-3-carboxamide (46)**

Yield 81%, white solid, mp: 243.5 °C; ^1^H NMR (500 MHz, DMSO-*d*_6_) δ 12.02 (s, 1H), 8.51 (d, *J* = 1.3 Hz, 1H), 8.15 (d, *J* = 2.8 Hz, 1H), 8.07 (t, *J* = 5.7 Hz, 1H), 7.97 (s, 1H), 7.55–7.58 (m, 2H), 7.47 (dd, *J* = 8.5, 1.6 Hz, 1H), 7.43 (dd, *J* = 9.4, 0.9 Hz, 1H), 7.36 (d, *J* = 0.9 Hz, 1H), 7.12–7.14 (m, 2H), 3.09 (t, *J* = 4.4 Hz, 4H), 2.53–2.60 (m, 4H), 2.32–2.41 (m, 2H), 1.87–2.04 (m, 2H), 1.72 (td, *J* = 14.0, 6.8 Hz, 2H); ^13^C NMR (500 MHz, DMSO-*d*_6_) δ 164.24, 160.29, 150.07, 149.58, 148.83, 138.26, 130.48, 128.26, 126.90, 125.11, 121.00, 118.59, 113.87, 112.32, 111.99, 110.20, 108.05, 103.07, 56.18, 53.43, 50.29, 40.65, 40.56, 40.48, 40.39, 40.32, 40.23, 40.15, 40.06, 39.98, 39.89, 39.73, 39.56, 37.60, 27.21; HRMS (FAB) calc. for C_26_H_26_N_6_O_3_, [M+H]^+^: 471.2145, found: 471.2144.

**5-Cyano-*N*-(3-(4-(2-((2,4-dimethylphenyl)thio)phenyl)piperazin-1-yl)propyl)-1*H*-indole-3-carboxamide (47)**

Yield 22%, white solid, mp: 217.7 °C; ^1^H NMR (500 MHz, DMSO-*d*_6_) δ 12.02 (d, *J* = 1.6 Hz, 1H), 8.51–8.54 (m, 1H), 8.16–8.19 (m, 1H), 8.08 (t, *J* = 5.7 Hz, 1H), 7.58 (d, *J* = 8.5 Hz, 1H), 7.47 (dd, *J* = 8.5, 1.6 Hz, 1H), 7.27–7.31 (m, 1H), 7.19 (d, *J* = 15.1 Hz, 1H), 7.03–7.08 (m, 3H), 6.83–6.89 (m, 1H), 6.33–6.35 (m, 1H), 3.28 (t, *J* = 6.6 Hz, 2H), 2.95–3.09 (m, 4H), 2.48–2.65 (m, 4H), 2.42 (s, 2H), 2.24–2.31 (m, 3H), 2.16–2.22 (m, 3H), 1.68–1.76 (m, 2H); ^13^C NMR (500 MHz, DMSO-*d*_6_) δ 164.27, 149.59, 142.18, 139.60, 138.40, 136.27, 133.87, 132.19, 130.50, 128.51, 127.91, 126.90, 126.42, 126.33, 126.14, 125.12, 124.76, 121.00, 120.54, 113.87, 112.00, 103.08, 56.21, 53.63, 51.70, 37.60, 27.11, 21.23, 20.62; HRMS (FAB) calc. for C_31_H_33_N_5_OS, [M+H]^+^: 524.2484, found: 524.2486.

***N*-(3-(4-(Benzo[*d*]isothiazol-3-yl)piperazin-1-yl)propyl)-5-cyano-1*H*-indole-3-carboxamide (48)**

Yield 43%, white solid, mp: 227.0 °C; ^1^H NMR (500 MHz, DMSO-*d*_6_) δ 12.03 (d, *J* = 1.9 Hz, 1H), 8.35–8.51 (m, 1H), 8.17 (dd, *J* = 11.8, 3.3 Hz, 1H), 8.05–8.10 (m, 1H), 8.01 (d, *J* = 9.1 Hz, 2H), 7.57–7.65 (m, 1H), 7.50–7.53 (m, 1H), 7.44–7.49 (m, 1H), 7.37–7.41 (m, 1H), 3.43 (s, 4H), 3.29 (t, *J* = 6.6 Hz, 2H), 2.60–2.76 (m, 4H), 2.47 (d, *J* = 1.9 Hz, 2H), 1.70–1.76 (m, 2H); ^13^C NMR (500 MHz, DMSO-*d*_6_) δ 164.29, 163.98, 152.52, 138.40, 130.50, 128.40, 127.86, 126.89, 126.42, 125.13, 124.94, 124.67, 121.59, 121.00, 113.88, 111.96, 103.08, 56.14, 55.42, 53.01, 50.02, 37.49, 27.07; HRMS (FAB) calc. for C_24_H_24_N_6_OS, [M+H]^+^: 445.1811, found: 445.1814.

**5-Cyano-*N*-(3-(4-(4-fluorobenzoyl)piperazin-1-yl)propyl)-1*H*-indole-3-carboxamide (49)**

Yield 29%, white solid, mp: 140.3 °C; ^1^H NMR (500 MHz, DMSO-*d*_6_) δ 12.02 (s, 1H), 8.50 (s, 1H), 8.14 (d, *J* = 1.8 Hz, 1H), 8.06 (t, *J* = 5.3 Hz, 1H), 7.57 (d, *J* = 8.3 Hz, 1H), 7.47 (dd, *J* = 8.5, 1.6 Hz, 1H), 7.41 (ddd, *J* = 11.9, 5.3, 3.2 Hz, 2H), 7.20–7.26 (m, 2H), 3.51 (d, *J* = 40.0 Hz, 2H), 3.25 (q, *J* = 6.4 Hz, 4H), 2.34 (t, *J* = 7.1 Hz, 6H), 1.62–1.74 (m, 2H); ^13^C NMR (500 MHz, DMSO-*d*_6_) δ 168.51, 164.23, 162.00, 138.39, 132.88, 130.48, 130.06, 129.99, 126.88, 126.42, 125.11, 120.99, 115.99, 115.81, 113.87, 111.96, 103.07, 55.98, 53.10, 47.89, 37.47, 27.17; HRMS (FAB) calc. for C_24_H_24_FN_5_O_2_, [M+H]^+^: 434.1992, found: 434.1988.

**5-Cyano-*N*-(3-(4-(3-(trifluoromethyl)benzoyl)piperazin-1-yl)propyl)-1*H*-indole-3-carboxamide (50)**

Yield 36%, white solid, mp: 142.8 °C; ^1^H NMR (500 MHz, DMSO-*d*_6_) δ 12.01 (s, 1H), 8.51–8.54 (m, 1H), 8.15–8.18 (m, 1H), 8.05 (t, *J* = 5.5 Hz, 1H), 7.56–7.61 (m, 1H), 7.46–7.50 (m, 1H), 7.35–7.41 (m, 1H), 7.16–7.22 (m, 1H), 7.11 (s, 1H), 7.00–7.05 (m, 1H), 3.28–3.38 (m, 2H), 3.14–3.18 (m, 4H), 2.48 (t, *J* = 4.6 Hz, 4H), 2.32–2.39 (m, 2H), 1.67–1.73 (m, 2H); ^13^C NMR (500 MHz, DMSO-*d*_6_) δ 164.23, 151.76, 138.39, 130.48, 130.46, 130.26, 126.90, 126.43, 126.07, 125.09, 123.90, 120.99, 119.15, 114.99, 113.86, 111.99, 111.29, 111.26, 103.08, 56.16, 53.12, 48.14, 37.58, 27.23; HRMS (FAB) calc. for C_25_H_24_F_3_N_5_O_2_, [M+H]^+^: 484.1960, found: 484.1956.

**5-Cyano-*N*-(3-(4-(2,3-dihydrobenzo[*b*][1,4]dioxine-2-carbonyl)piperazin-1-yl)propyl)-1*H*-indole-3-carboxamide (51)**

Yield 82%, white solid, mp: 143.1 °C; ^1^H NMR (500 MHz, DMSO-*d*_6_) δ 11.94 (d, *J* = 65.6 Hz, 1H), 8.48–8.51 (m, 1H), 8.16 (d, *J* = 15.1 Hz, 1H), 8.00–8.06 (m, 1H), 7.52–7.62 (m, 1H), 7.42–7.52 (m, 1H), 6.83–6.90 (m, 1H), 6.68–6.82 (m, 3H), 5.13–5.26 (m, 1H), 4.27–4.34 (m, 1H), 4.13 (dd, *J* = 11.6, 6.6 Hz, 1H), 3.38–3.57 (m, 4H), 3.27 (q, *J* = 6.6 Hz, 2H), 2.29–2.43 (m, 6H), 1.65–1.70 (m, 2H); ^13^C NMR (500 MHz, DMSO-*d*_6_) δ 165.15, 164.24, 143.56, 143.37, 138.39, 130.48, 126.89, 126.42, 125.12, 121.98, 121.87, 120.99, 117.53, 117.38, 113.87, 111.97, 103.08, 69.91, 65.24, 55.97, 53.54, 52.95, 45.59, 41.99, 37.48, 27.16; HRMS (FAB) calc. for C_26_H_27_N_5_O_4_, [M+H]^+^: 474.2141, found: 474.2135.

**5-Cyano-*N*-(2-(4-(6-fluorobenzo[*d*]isoxazol-3-yl)piperidin-1-yl)ethyl)-1*H*-indole-3-carboxamide (52)**

Yield 88%, white solid, mp: 208.9 °C; ^1^H NMR (500 MHz, DMSO-*d*_6_) δ 12.02 (s, 1H), 8.51 (d, *J* = 1.6 Hz, 1H), 8.16 (d, *J* = 2.8 Hz, 1H), 7.95–8.00 (m, 2H), 7.64 (dd, *J* = 9.1, 1.9 Hz, 1H), 7.58 (dd, *J* = 8.5, 0.6 Hz, 1H), 7.48 (dd, *J* = 8.5, 1.6 Hz, 1H), 7.22 (td, *J* = 9.1, 2.2 Hz, 1H), 3.39–3.47 (m, 2H), 3.12 (s, 1H), 3.01 (d, *J* = 8.2 Hz, 2H), 2.48–2.58 (m, 2H), 2.18 (s, 2H), 1.99–2.04 (m, 2H), 1.84 (t, *J* = 10.8 Hz, 2H); ^13^C NMR (500 MHz, DMSO-*d*_6_) δ 165.18, 164.26, 163.59, 163.15, 138.44, 130.62, 126.84, 126.35, 125.14, 124.37, 124.28, 120.96, 117.77, 113.91, 113.13, 112.89, 111.87, 103.13, 97.97, 97.71, 57.89, 53.55, 37.05, 33.80, 30.58; HRMS (FAB) calc. for C_23_H_21_FN_6_O_2,_ [M+H]^+^: 432.1836, found: 432.1834.

***N*-(2-(4-(5-Chloro-2-oxo-2,3-dihydro-1*H*-benzo[*d*]imidazol-1-yl)piperidin-1-yl)ethyl)-5-cyano-1*H*-indole-3-carboxamide (53)**

Yield 90%, white solid, mp: 266.7 °C; ^1^H NMR (500 MHz, DMSO-*d*_6_) δ 12.06 (s, 1H), 10.99 (s, 1H), 8.51 (d, *J* = 0.9 Hz, 1H), 8.17 (d, *J* = 2.5 Hz, 1H), 8.03 (s, 1H), 7.59 (dd, *J* = 8.5, 0.9 Hz, 1H), 7.48 (dd, *J* = 8.5, 1.6 Hz, 1H), 7.15 (d, *J* = 8.5 Hz, 1H), 6.91–6.95 (m, 2H), 4.11 (s, 1H), 3.37–3.49 (m, 2H), 3.03–3.13 (m, 2H), 2.48–2.60 (m, 2H), 2.29 (q, *J* = 11.2 Hz, 2H), 2.13 (d, *J* = 7.5 Hz, 2H), 1.61–1.72 (m, 2H); ^13^C NMR (500 MHz, DMSO-*d*_6_) δ 164.28, 154.20, 138.42, 130.65, 129.94, 128.68, 126.85, 126.40, 125.27, 125.14, 120.97, 120.52, 113.92, 111.93, 110.30, 109.21, 103.13, 57.59, 53.26, 50.82, 37.08, 29.06; HRMS (FAB) calc. for C_23_H_22_ClN_7_O_2_, [M+H]^+^: 463.1649, found: 463.1659.

**5-Cyano-*N*-(3-(4-(6-fluorobenzo[*d*]isoxazol-3-yl)piperidin-1-yl)propyl)-1*H*-indole-3-carboxamide (54)**

Yield 96%, white solid, mp: 224.1 °C; ^1^H NMR (500 MHz, DMSO-*d*_6_) δ 12.02 (s, 1H), 8.51–8.54 (m, 1H), 8.16–8.19 (m, 1H), 8.07 (t, *J* = 5.3 Hz, 1H), 7.94–8.01 (m, 1H), 7.62–7.69 (m, 1H), 7.55–7.58 (m, 1H), 7.36–7.53 (m, 1H), 7.22 (td, *J* = 8.9, 2.1 Hz, 1H), 3.27 (d, *J* = 6.9 Hz, 2H), 3.11 (t, *J* = 11.1 Hz, 1H), 2.98 (s, 2H), 2.32–2.41 (m, 2H), 2.10 (s, 2H), 1.93–2.01 (m, 2H), 1.78–1.90 (m, 2H), 1.68–1.74 (m, 2H); ^13^C NMR (500 MHz, DMSO-*d*_6_) δ 165.14, 164.25, 163.58, 163.47, 163.17, 161.73, 138.40, 130.48, 126.89, 126.43, 125.09, 124.35, 124.25, 120.99, 117.77, 113.87, 113.10, 112.90, 111.99, 103.07, 97.96, 97.75, 56.27, 53.47, 37.55, 33.66, 30.41, 26.87; HRMS (FAB) calc. for C_24_H_23_FN_6_O_2_, [M+H]^+^: 446.1992, found: 446.2002.

***N*-(3-(4-(5-Chloro-2-oxo-2,3-dihydro-1*H*-benzo[*d*]imidazol-1-yl)piperidin-1-yl)propyl)-5-cyano-1*H*-indole-3-carboxamide (55)**

Yield 82%, white solid, mp: 197.8 °C; ^1^H NMR (500 MHz, DMSO-*d*_6_) δ 12.02 (s, 1H), 10.98 (s, 1H), 8.47–8.55 (m, 1H), 8.16 (d, *J* = 2.2 Hz, 1H), 8.03–8.08 (m, 1H), 7.57–7.62 (m, 1H), 7.47 (dd, *J* = 8.5, 1.6 Hz, 1H), 7.16 (d, *J* = 8.5 Hz, 1H), 6.96 (dd, *J* = 9.9, 1.7 Hz, 2H), 4.10 (t, *J* = 11.9 Hz, 1H), 3.27 (s, 2H), 2.93–3.01 (m, 2H), 2.40 (s, 2H), 2.25–2.32 (m, 2H), 1.84–2.04 (m, 2H), 1.68–1.75 (m, 2H), 1.61–1.63 (m, 2H); ^13^C NMR (500 MHz, DMSO-*d*_6_) δ 164.25, 154.20, 138.40, 130.49, 130.08, 128.70, 126.89, 126.43, 125.28, 125.11, 120.99, 120.57, 113.88, 111.99, 110.29, 109.21, 103.07, 55.92, 53.20, 50.97, 37.55, 29.15, 27.36; HRMS (FAB) calc. for C_24_H_24_ClN_7_O_2_, [M+H]^+^: 477.1806, found: 477.1815.

### 3.2. In Vitro Receptor Assay

The screening of pain-related receptors for the mechanism of action study of compound **29** was conducted by outsourcing the experiment to Eurofins (https://www.eurofins.com, accessed on 30 July 2024). Methods employed in this study have been adapted from the scientific literature to maximize reliability and reproducibility. Reference standards were run as an integral part of each assay to ensure the validity of the results obtained. The assays were performed under the conditions described below for each receptor. Where presented, IC_50_ values were determined by a non-linear, least squares regression analysis using MathIQTM (ID Business Solutions Ltd., Woking, Surrey, UK). Where inhibition constants (K_i_) are presented, the Ki values were calculated using the equation of Cheng and Prusoff [22] using the observed IC_50_ of the tested compound, the concentration of radioligand (*) employed in the assay, and the historical values for the K_D_ of the ligand (obtained experimentally at Eurofins Panlabs, Inc.). Where presented, the Hill coefficient (nH), defining the slope of the competitive binding curve, was calculated using MathIQTM. Hill coefficients significantly different than 1.0 may suggest that the binding displacement does not follow the laws of mass action with a single binding site.

#### 3.2.1. Adrenergic α_2A_

Source: human recombinant CHO-Ki cells, vehicle: 1.0% DMSO, incubation time/temp: 60 min @ 25 °C, incubation buffer: 50 mM Tris-HCl, pH 7.4, 1 mM EDTA, K_d_: 1.50 nM*, ligand: 1.50 nM [^3^H] Rauwolscine, non-specific ligand: 10.0 μM WB-4101, specific binding: 95%*, quantitation method: radioligand binding, significance criteria: ≥50% of max stimulation or inhibition, B_max_: 16.0 pmole/mg protein*

#### 3.2.2. Dopamine D_2L_

Source: human recombinant CHO cells, vehicle: 1.0% DMSO, incubation time/temp: 2 h @ 25 °C, incubation buffer: 50 mM Tris-HCl, pH 7.4, 1.4 mM ascorbic acid, 0.001% BSA, 150 mM NaCl, Kd: 0.08 nM*, ligand: 0.16 nM [^3^H] Spiperone, non-specific ligand: 10.0 μM Haloperidol, specific binding: 85%* quantitation method: radioligand binding, significance criteria: ≥50% of max stimulation or inhibition, B_max_: 0.48 pmole/mg protein*

#### 3.2.3. Serotonin 5-HT_2A_

Source: human recombinant CHO-Ki cells, vehicle: 1.0% DMSO, incubation time/temp: 60 min @ 25 °C, incubation buffer: 50 mM Tris-HCl, pH 7.4, Kd: 0.2 nM*, ligand: 0.5 nM [^3^H] Ketanserin, non-specific ligand: 1.0 μM Mianserin, specific binding: 90%*, quantitation method: radioligand binding, significance criteria: ≥50% of max stimulation or inhibition, B_max_: 0.51 pmole/mg protein*

#### 3.2.4. Transporter, Dopamine (DAT)

Source: human recombinant CHO-S cells, vehicle: 1.0% DMSO, incubation time/temp: 3 h @ 4 °C, incubation buffer: 50 mM Tris-HCl, pH 7.4, 100 mM NaCl, 1 μM Leupeptin, 10 μM PMSF, Kd: 0.58 nM*, Ligand: 0.15 nM [^125^I] RTI-55, non-specific ligand: 10.0 μM Nomifensine, specific binding: 90%*, quantitation method: radioligand binding, significance criteria: ≥50% of max stimulation or inhibition, B_max_: 0.047 pmole/mg protein*

#### 3.2.5. Transporter, Norepinephrine (NET)

Source: human recombinant MDCK cells, vehicle: 1.0% DMSO, incubation time/temp: 3 h @ 4 °C, incubation buffer: 50 mM Tris-HCl, pH 7.4, 100 mM NaCl, 1 μM Leupeptin, 10 μM PMSF, Kd: 0.024 nM*, ligand: 0.2 nM [^125^I] RTI-55, non-specific ligand: 10.0 μM Desipramine, specific binding: 75%*, quantitation method: radioligand binding, significance criteria: ≥50% of max stimulation or inhibition, B_max_: 2.50 pmole/mg protein*

#### 3.2.6. Transporter, Serotonin (SERT)

Source: human recombinant HEK-293 cells, vehicle: 1.0% DMSO, incubation time/temp: 60 min @ 25 °C, incubation buffer: 50 mM Tris-HCl, pH 7.4, 120 mM NaCl, 5 mM KCl, Kd: 0.078 nM*, ligand: 0.4 nM [^3^H] Paroxetine, non-specific ligand: 10.0 μM Imipramine, specific binding: 95%*, quantitation method: radioligand binding, significance criteria: ≥50% of max stimulation or inhibition, B_max_: 4.40 pmole/mg protein*

### 3.3. In Vivo Assay

#### 3.3.1. Animals

ICR male mice weighing 23–25 g was purchased from Samtako Korea (Osan, Republic of Korea). ICR mice were housed four per cage in a room with 12 h light–dark cycles. The temperature and relative humidity of the room were maintained at 22 ± 2 °C and 50 ± 5%, respectively. Food and water were available ad libitum. The procedures for animal testing were approved by Medifron Animal Care and Use Committees (Approval number; Medifron 2019-9). Efforts were made to minimize animal suffering and to reduce the number of animals used.

#### 3.3.2. Formalin Model Test

Mice were randomly assigned to five groups (four per group) and single-dose drugs were administered by intraperitoneal injection. The formalin-induced licking paw test was modified from the method described by Dubuisson and Dennis [14]. Each mouse was acclimated to an acrylic observation chamber for at least 30 min before the injection of formalin. Twenty microliters of 2% formalin were injected subcutaneously into the right side of the hind paw. Each mouse was then placed in an individual clear plastic observational chamber (15 × 15 × 15 cm, volume = 3375 cm^2^ or 3.375 L), and the pain response was recorded for a period of 30 min. The summation of time (in seconds) spent in licking and biting responses of the injected paw during each 5 min block was measured as an indicator of the pain response. The first period (early phase) was recorded 0–5 min after the injection of formalin, and the second period (late phase) was recorded 20–30 min after the injection. The test compound was administered intraperitoneally 30 min before the formalin injection at three different doses: 0.1, 1, 5, and 10 mg/kg. The vehicle was DMSO/Cremophor EL/distilled water (10/10/80). The data were expressed as the mean ± standard error of the mean (SEM). Statistical analysis was assessed by one-way analysis of variance (ANOVA) with Bonferroni’s post hoc test. Statistical significance was set at *p*-value < 0.05, with values of *p* <0.001 considered highly significant.

#### 3.3.3. Spinal Nerve Ligation (SNL) Model Test

Spinal nerve ligation (SNL) was performed following the method described by Kim and Chung [20]. Rats were anesthetized with 4% isoflurane in 95% oxygen, and anesthesia was maintained throughout the procedure. After sterilization, the left muscles over the L4-S2 spinal cord region were carefully removed. The L5 transverse process was resected to expose the L4-L6 spinal nerves. The L5 spinal nerve was identified, gently separated from surrounding tissues, and tightly ligated with 6-0 black silk thread. Once hemostasis was achieved, the wound was closed in layers using stainless steel wound clips. Following the surgery, the rats were returned to their cages and monitored during recovery.

Behavioral signs indicative of neuropathic pain (mechanical allodynia) were assessed in all rats for two weeks postoperatively. The paw withdrawal threshold (PWT) was measured using von Frey filaments (Stoelting, Wood Dale, IL, USA) and the up–down method [21]. A series of eight von Frey filaments (0.4–15 g) were applied perpendicularly to the plantar surface of the hind paw for 5 s, ensuring the filament bent during application. A brisk withdrawal or paw flinching was recorded as a positive response. If a positive response occurred, the next trial used a less stiff filament; if no withdrawal or licking was observed, a stiffer filament was applied. The cut-off threshold was set at 15 g, meaning the PWT was recorded as 15 g if no withdrawal or licking response was observed with the application of a 15 g von Frey filament. PWT measurements were obtained prior to drug administration and at 1, 3, and 5 h post-administration.

Rats exhibiting mechanical allodynia were randomly assigned to three groups on the 14th day after SNL surgery. The three experimental groups—vehicle, 50 mg/kg of compound **29**, and 100 mg/kg of compound **29**—received intraperitoneal injections of compound **29** formulated in a DMSO/Cremophor EL/distilled water mixture (10:10:80).

All data are expressed as mean ± standard error of the mean (SEM). The normality of the data was assessed using the Shapiro–Wilk test. Data analysis was conducted using an independent samples t-test, with statistical significance set at *p* < 0.05. All statistical analyses were performed using GraphPad Prism software (version 9.3).

#### 3.3.4. Statistical Analysis

The results from the PC12 differentiation assay were reported as mean ± SEM of three different experiments and were evaluated using ANOVA followed by Dunnett’s post hoc test. The results from the water permeability assay were expressed as means ± SEM of 4–15 single shots (time course curves) for each of the 4–6 different experiments and were analyzed by ANOVA, followed by the Newman–Keuls Q test. The results from the in vivo toxicity in the zebrafish model were reported as the mean of the values assessed using a sample score sheet, as reported by Brannen KC and colleagues. Data were checked for normality using the Shapiro–Wilk test. The formalin assay results are presented as the mean ± SEM for 8 mice per group and were analyzed using one-way analysis of variance (ANOVA) with Bonferroni’s post hoc test. In addition, the data from Chung’s neuropathic pain model are expressed as the mean ± SEM and were analyzed using Student’s t-test. The data were considered to be statistically significant if *p* < 0.05 (*), *p* < 0.01 (**), *p* < 0.001 (***), and *p* < 0.0001 (****). Statistical analysis was performed using GraphPad Prism Software (version 9.3).

### 3.4. Pharmacokinetic Study

#### 3.4.1. Metabolic Stability Assay

Human (or mouse) liver microsomes (0.5 mg/mL) (human: Corning #452117, mouse: Corning #452501; Corning, NY, USA) were mixed with 0.1 M potassium phosphate buffer (pH 7.4) (Corning, #451201) and the test compound at a concentration of 1 μM. The mixture was pre-incubated at 37 °C for 5 min, followed by the addition of NADPH Regeneration System solution (Promega, V9510; Madison, WI, USA) and further incubation at 37 °C for 30 min. To terminate the reaction, an acetonitrile solution containing the internal standard chlorpropamide (TRC, Toronto Research Chemicals, Toronto, ON, Canada; C424800) was added, and the mixture was centrifuged at 15,000 rpm for 5 min at 4 °C. The supernatant was injected into an LC-MS/MS system to analyze the substrate drugs and evaluate the metabolic stability of the compound.

The remaining substrate was quantified using the Nexera XR system (Shimadzu, Japan) and TSQ Vantage mass spectrometer (Thermo, Waltham, MA, USA). The HPLC system utilized a Kinetex C18 column (2.1 mm × 100 mm, 2.6 μm particle size, Phenomenex, Torrance, CA, USA). The mobile phases consisted of distilled water containing 0.1% formic acid (A) and acetonitrile containing 0.1% formic acid (B), with gradient elution conditions. Metabolites were analyzed using the Multiple Reaction Monitoring (MRM) quantification mode in the Xcalibur software (version 4.4). The experiment was performed in duplicate with variability within 15%. Results were calculated as % remaining by comparing the test compound quantified after a 30 min reaction to the initial value at 0 min. To validate experimental accuracy, a positive control, verapamil (1 μM) (TRC, V125000), was tested in human microsomes only, and the results met the internal quality control criteria of 15% (±10%).

#### 3.4.2. BBB PAMPA Assay

The BBB PAMPA assay begins with preparing the donor plate, where a phospholipid mixture (e.g., lecithin in dodecane) is applied to the filter membrane to mimic the blood–brain barrier. Test compounds are dissolved in an appropriate solvent, typically DMSO, and diluted with a buffer (e.g., PBS at pH 7.4) to the desired concentration, then added to the donor wells. The acceptor plate is filled with a buffer simulating the brain environment, such as phosphate buffer or artificial cerebrospinal fluid. The donor plate is placed on the acceptor plate to form a sandwich structure, ensuring alignment of the wells, and incubated at 25–37 °C for 4–6 h. After incubation, samples from both donor and acceptor wells are collected and analyzed to determine compound concentrations using LC-MS/MS or UV–visible spectrophotometry. The permeability coefficient (Pe) is calculated from the data to assess the compound’s BBB permeability. Positive and negative controls, such as propranolol and atenolol, are included to validate the assay conditions.

#### 3.4.3. In Vivo Pharmacokinetic Study

Male Sprague–Dawley rats (n = 3 per group, weight 200–250 g) were fasted overnight but had free access to water. All animal experiments were conducted in accordance with institutional guidelines. For intraperitoneal (IP) administration, compound **29** was dissolved in saline and DMSO, while for oral (PO) administration, the compound was suspended in 0.5% carboxymethyl cellulose (CMC). All solutions were freshly prepared and filtered before administration to ensure homogeneity. Compound **29** was administered at 5 mg/kg via intraperitoneal injection or at 10 mg/kg by oral gavage. Blood samples (approximately 0.3 mL) were collected at 0.25, 0.5, 1, 2, 4, 6, 8, and 24 h post-dose to capture key pharmacokinetic parameters such as Cmax, Tmax, and the elimination phase. Blood was collected into EDTA-coated tubes and centrifuged at 2000× *g* for 10 min at 4 °C to separate plasma. Plasma samples were stored at −80 °C until analysis. Before analysis, plasma samples were thawed on ice, and proteins were precipitated using acetonitrile containing an internal standard. The mixture was centrifuged, and the supernatant was collected for analysis.

Plasma concentrations of compound **29** were determined using a validated LC-MS/MS method. The analysis was conducted on a Nexera XR HPLC system (Shimadzu, Japan) coupled with a TSQ Vantage mass spectrometer (Thermo, Waltham, MA, USA), using a Kinetex C18 column (2.1 × 100 mm, 2.6 µm particle size, Phenomenex, Torrance, CA, USA). A gradient elution of 0.1% formic acid in water (A) and 0.1% formic acid in acetonitrile (B) was employed. Quantification was performed in Multiple Reaction Monitoring (MRM) mode using Xcalibur software (version 4.4). Pharmacokinetic parameters, including AUC_last_, AUC_inf_, C_max_, T_max_, t_1/2_, Cl/F, and V_d_, were calculated using non-compartmental analysis (NCA) with Phoenix WinNonlin software 8.5.2 (Certara, Radnor, PA, USA).

### 3.5. Toxicity Study

#### 3.5.1. Cytotoxicity Assay

HT-22 (mouse hippocampal cells) were cultured in Dulbecco’s Modified Eagle’s Medium (DMEM, GIBCO) supplemented with 10% (*v*/*v*) fetal bovine serum and antibiotics (100 µg/mL penicillin/streptomycin mix) in a humidified atmosphere at 37 °C with 5% CO_2_. A total of 5000 HT-22 cells per well were seeded into a clear 96-well plate one day prior to the assay. On the day of the experiment, the medium was removed, and the cells were treated with 25 µL of each compound at a concentration of 10 µM. The plates were incubated at 37 °C for 18 h. Subsequently, 15 µL of 3-[4,5-dimethylthiazol-2-yl]-2,5-diphenyltetrazolium bromide (MTT) solution (5 mg/mL) was added to each well, and the plates were incubated for an additional 3 h. The resulting formazan crystals were dissolved in dimethyl sulfoxide (DMSO), and the absorbance at 570 nm was measured using a microplate reader (Sunrise, TECAN). Optical density (OD) values from each well were normalized by subtracting the vehicle control, and cell viability was calculated by considering the signal from the vehicle control as 100%.

#### 3.5.2. hERG Channel Inhibition Assay

The hERG Fluorescence Polarization Assay Kit (Invitrogen, Carlsbad, CA, USA; PV5365) was used as the test material. The procedure involved adding the test substance or E-4031 as a positive control to a mixture of Predictor™ hERG Membrane and Predictor™ hERG Tracer solution, followed by incubation for 4 h. Fluorescence intensity at each concentration was measured using a Synergy Neo microplate reader (Biotek, Winooski, VT, USA) with excitation at 530 nm and emission at 590 nm. For data analysis, IC_50_ values were calculated based on polarization [mP] values derived from the fluorescence intensity. The safety threshold for hERG channel inhibition was set at 10 µM, and the potential for hERG channel inhibition was assessed accordingly.

#### 3.5.3. Cytochrome P450 (CYP) Inhibition Assay

Human liver microsomes (0.25 mg/mL) (Corning, #452117) and 0.1 M potassium phosphate buffer (pH 7.4) (Corning, #451201) were mixed with a cocktail of substrate drugs for five drug-metabolizing enzymes and the test compound at concentrations of 0 and 10 μM. The mixture was pre-incubated at 37 °C for 5 min. Subsequently, the NADPH Regeneration System solution (Promega, V9510) was added, and the reaction was incubated at 37 °C for 15 min. To terminate the reaction, an acetonitrile solution containing the internal standard terfenadine (Sigma Aldrich, St. Louis, MO, USA, T9562) was added. The mixture was centrifuged at 15,000 rpm for 5 min at 4 °C, and the supernatant was injected into an LC-MS/MS system to simultaneously analyze the metabolites of the substrate drugs (CYP1A2: Phenacetin, CYP2C9: Diclofenac, CYP2C19: S-Mephenytoin, CYP2D6: Dextromethorphan, CYP3A4: Midazolam) and evaluate the inhibitory potential of the test compound on drug-metabolizing enzymes.

The metabolites generated from each CYP isoenzyme-specific substrate were analyzed using a Nexera XR system (Shimadzu, Japan) and a TSQ Vantage mass spectrometer (Thermo, Waltham, MA, USA). The HPLC system utilized a Kinetex C18 column (2.1 × 100 mm, 2.6 μm particle size, Phenomenex, Torrance, CA, USA). The mobile phases were distilled with water containing 0.1% formic acid (A) and acetonitrile containing 0.1% formic acid (B), with a gradient elution method. The metabolites were quantified using the Multiple Reaction Monitoring (MRM) mode in the Xcalibur software (version 4.4).

The experiment was performed in duplicate, and the variability was within 15%. The results were expressed as the percentage activity of each of the CYP isoenzyme’s metabolites compared to the control (without inhibitor) after a 15 min reaction. To validate the accuracy of the experiment, a positive control (ketoconazole, a selective CYP3A4 inhibitor, 0.1 μM) was included, and the results met the internal quality control criteria with a value of 25% (±10%).

## 4. Conclusions

Pain is a multifaceted experience influenced by peripheral, central, immune, and psychological factors. Targeting multiple pathways offers a holistic approach, improving efficacy, minimizing side effects, and reducing tolerance compared to single-target drugs, but it also carries the risk of adverse effects due to interactions with additional targets. The in vivo-guided approach is a promising tool for discovering multitarget analgesics. This study used this to identify a novel analgesic targeting key pathways in pain perception, transmission, and regulation.

A new scaffold (**3**) inspired by the pharmacophores of opiranserin and vilazodone has been designed and the synthesized analogs were screened for the second phase inhibition in the formalin test, among which compound **29** was selected for further investigation.

In the formalin model, compound **29** exhibited a high potency with an ED_50_ value of 0.78 mg/kg in the second phase with a tendency of concentration-dependent inhibition during the first phase. In the SNL neuropathic pain model, it demonstrated dose-dependent analgesic effects by increasing the withdrawal threshold with 24 and 45% MPE at 50 and 100 mg/kg, respectively. Extensive mechanism studies suggested that its analgesic activity may be linked to its strong triple uptake inhibition, particularly at DAT and SERT, along with 5-HT2A antagonism. However, the direct involvement of these mechanisms in the observed antinociceptive effects has not been definitively demonstrated and requires further investigation.

In the BBB PAMPA experiment, it showed a high BBB permeability suitable for CNS drugs. The in vivo pharmacokinetics study in rats showed that IP administration provided a five-fold greater exposure and two-fold higher stability compared to oral administration. Following IP administration, the half-life was 3.49 h with a T_max_ of 2.83 h, while po administration resulted in a half-life of 1.67 h and a T_max_ of 1.50 h. For the in vitro toxicity study, it was nontoxic to the HT-22 normal neuronal cell but potential hERG inhibition with 52.7% inhibition at 10 μM. Additionally, in the CYP inhibition study, it displayed strong inhibition of CYP3A4, while showing minimal inhibition of other tested isozymes.

Overall, compound **29** is a novel and potent analgesic identified through an in vivo-guided approach with a multitarget mechanism. Further optimization is in progress to minimize the observed side effects.

## Figures and Tables

**Table 1:**
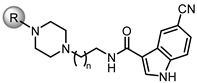
5-Cyano-*N*-(2-(4-arylpiperazin-1-yl)ethyl/propyl)-indole-3-carboxamide analogs.

R	n = 1	% Inhibition	n = 2	% Inhibition
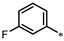	**20**	3.1	**36**	NE
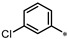	**21**	90.6	**37**	81.4
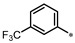	**22**	94.6	**38**	37.1
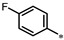	**23**	35.8	**39**	NE ^a^
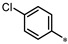	**24**	1.6	**40**	4.7
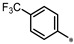	**25**	19.8	**41**	16.5
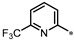	**26**	48.6	**42**	45.7
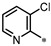	**27**	48.0	**43**	45.3
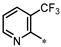	**28**	NE	**44**	3.7
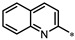	**29**	98	**45**	96.5
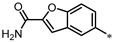	**30**	23.8	**46**	49.2
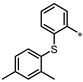	**31**	75.0	**47**	NE
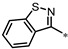	**32**	14.3	**48**	NE
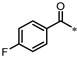	**33**	24.2	**49**	20.3
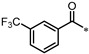	**34**	2.1	**50**	2.9
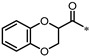	**35**	0.4	**51**	27.3

^a^ NE: no effect.

**Table 2:**
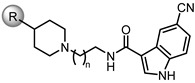
5-Cyano-*N*-2(3)-(4-arylpiperidin-1-yl)ethyl(propyl)-indole-3-carboamide.

R	n = 1	% Inhibition	n = 2	% Inhibition
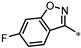	**52**	46.7	**54**	NE
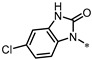	**53**	14.3	**55**	24.0

**Table 3:** ED_50_ values of the selected compounds in the formalin model.

Compound	% Inhibition	ED_50_
**21**	90.6	2.3
**22**	94.6	1.3
**29**	98	0.78
**31**	75	2.66
**37**	81.4	3.2
**45**	96.5	0.93

**Table 4:** IC_50_ values of compound **29** for the receptors with the highest potency.

	IC_50_ (µM)	K_i_ (µM)	n_H_ ^a^
**Transporter, Dopamine (DAT)**			
*Vanoxerine*	1.08	0.86	1.01
**29**	0.057	0.046	0.88
**Transporter, Norepinephrine (NET)**			
*Desipramine*	1.09	1.08	0.84
**29**	0.38	0.38	0.73
**Transporter, Serotonin (SERT)**			
*Fluoxetine*	10.1	1.64	0.74
**29**	0.78	0.13	0.84
**Adrenergic α_2A_**			
*Yohimbine*	2.08	1.04	0.88
**29**	8.22	4.11	1.02
**Dopamine D_2L_**			
*Spiperone*	0.12	0.041	0.71
**29**	5.54	1.85	0.86
**Serotonin 5-HT_2A_**			
*Ketanserin*	0.81	0.23	0.99
**29**	0.52	0.15	0.88

^a^ n_H_: Hill coefficient, n = 1 non-cooperative.

**Table 5:** In vitro pharmacokinetic profile of compound **29**.

Metabolic stability ^a^	51.2% (mouse), 55.6% (human)
BBB PAMPA	Log Pe = 4.5

^a^ % remaining for 30 min.

**Table 6:** Pharmacokinetics parameters of compound **29** following intraperitoneal and oral administration in Sprague–Dawley rats.

PK Parameters	Unit	IP	PO
Dose	mg/kg	5	10
AUC_last_	ng·h/mL	1030	407
AUC_inf_	ng·h/mL	1381	430
C_max_	ng/mL	208	113
T_max_	h	2.83	1.50
Cl/F	L/h/kg	3.88	24.04
V_d_	L/kg	17.9	57.4
t_1/2_	h	3.49	1.67

**Table 7:** hERG inhibition of compound **29**.

FP Assay	Inhibition, % ^a^
**29**	52.7
E-4031	100

^a^ % of inhibition at 10 µM.

**Table 8:** CYP isozyme inhibition of compound **29**.

	CYP1A2	CYP3A4	CYP2C9	CYP2C19	CYP2D6
**29** ^a^	0	78	47	43	11
Ketoconazole ^b^	0	68	3.2	0	0

^a^ % of inhibition at 10 µM; ^b^ CYP3A4 inhibitor (IC_50_ = 0.1 µM).

## Data Availability

Data are contained within the article.
